# Short salsalate administration affects cell proliferation, metabolism, and inflammation in polycystic kidney disease

**DOI:** 10.1016/j.isci.2023.108278

**Published:** 2023-10-19

**Authors:** Anish A. Kanhai, Elena Sánchez-López, Thomas B. Kuipers, Jan B. van Klinken, Kyra L. Dijkstra, Inge van der Veen, Hans J. Baelde, Xuewen Song, York Pei, Hailiang Mei, Wouter N. Leonhard, Oleg A. Mayboroda, Dorien J.M. Peters

**Affiliations:** 1 Department of Human Genetics, Leiden University Medical Center, Leiden, the Netherlands; 2 Center for Proteomics and Metabolomics, Leiden University Medical Center, Leiden, the Netherlands; 3 Sequencing Analysis Support Core, Department of Biomedical Data Sciences, Leiden University Medical Center, Leiden, the Netherlands; 4 Laboratory Genetic Metabolic Diseases of Pathology, Amsterdam UMC, University of Amsterdam, Amsterdam, the Netherlands; 5 Core Facility Metabolomics, Amsterdam UMC, University of Amsterdam, Amsterdam, the Netherlands; 6 Department of Pathology, Leiden University Medical Center, Leiden, the Netherlands; 7 Division of Nephrology, University Health Network and University of Toronto, Toronto, ON, Canada

**Keywords:** Biological sciences, Metabolomics, Omics, Transcriptomics

## Abstract

Metabolic reprogramming is a driver of autosomal dominant polycystic kidney disease (ADPKD) progression and a potential therapeutic intervention route. We showed before that the AMP-associated protein kinase (AMPK) activator salsalate attenuates cystic disease progression. Here, we aim to study the early, direct effects of short salsalate treatment in adult-onset conditional *Pkd1* deletion mice. Cystic mice were treated with salsalate for two weeks, after which NMR metabolomics and RNA sequencing analyses were performed. *Pkd1* deletion resulted in clear metabolomic dysregulation. Short salsalate treatment has small, but significant, effects, reverting acetylcarnitine and phosphocholine concentrations back to wildtype levels, and showing associations with altered purine metabolism. RNA sequencing revealed that short salsalate treatment, next to restoring energy metabolism toward wildtype levels, also affects cell proliferation and inflammation, in PKD. We show that salsalate positively affects major dysregulated processes in ADPKD: energy metabolism, cell proliferation, and inflammation, providing more insights into its working mechanisms.

## Highlights

• Salsalate changes acetylcarnitine, phosphocholine, and tyrosine levels in PKD
• Short salsalate treatment reverts Warburg-like metabolic reprogramming in PKD
• RNA sequencing shows salsalate reduces proliferation and inflammation in PKD
• Combined results confirm the versatility of salsalate as a potential PKD treatment

## Graphical abstract

**Figure:**
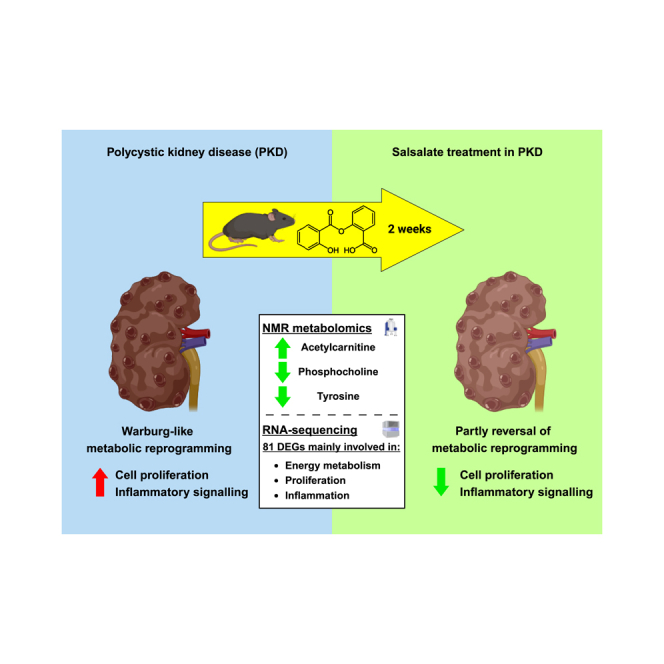

## Introduction

Autosomal dominant polycystic kidney disease (ADPKD) is the most common inherited kidney disease, affecting around 1 in 1,000 individuals.^1^ The majority of cases are caused by mutations in either the *PKD1* (∼85%) or *PKD2* (∼15%) gene, encoding the proteins polycystin-1 (PC-1) or polycystin-2 (PC-2), respectively.^2^^,^^3^ The exact working mechanism of these proteins remains elusive, but it is known that PC-1 and PC-2 form heterotetramers with 1:3 stoichiometry, functioning as ion channels.^4^^,^^5^^,^^6^^,^^7^ The ion channel function is directly affected by PC-1 through its contribution to the channel pore. PC-2 itself can also form homotetramers, which function as non-selective cation channels, distinctive from the PC-1/PC-2 heterotetramers.^7^^,^^8^^,^^9^

ADPKD is characterized by the formation of fluid-filled cysts, which increase over time in size and number. This puts the surrounding renal structure under stress, resulting in local injury and fibrosis, impairing kidney function.^10^ Consequently, a plethora of cellular changes are observed in the cystic epithelium, which go together with intracellular signaling network dysregulation.^1^^,^^10^ Of note are findings that cystic cells and tissues undergo Warburg-like metabolic reprogramming, in which aerobic glycolysis is elevated,^11^ while oxidative phosphorylation is reduced.^12^^,^^13^ In addition, changes in lipid metabolism,^12^^,^^14^ amino acid metabolism,^15^^,^^16^^,^^17^ and mitochondrial morphology and function^13^^,^^18^^,^^19^ have been reported. Therapeutic and dietary interventions based on these findings have proven to affect PKD progression in preclinical models.^20^^,^^21^^,^^22^^,^^23^^,^^24^^,^^25^

Currently, the vasopressin V2 receptor antagonist tolvaptan is the only approved drug for ADPKD treatment, but it is only suitable or approved for subsets of patients and has side effects like polyuria and liver toxicity.^26^^,^^27^ Therefore, the search for new therapeutic interventions for ADPKD continues and has mostly been focused on reversing dysregulated intracellular signaling effects. Preclinical studies have identified AMP-associated protein kinase (AMPK) as an interesting therapeutic target for ADPKD.^20^^,^^25^^,^^28^^,^^29^^,^^30^^,^^31^ Acting as a central cellular energy sensor, AMPK regulates various metabolic processes, including many dysregulated ones in ADPKD. We found that a long treatment with the direct AMPK activator salsalate attenuates cystic kidney disease at clinically relevant doses in an adult-onset inducible kidney-specific *Pkd1* deletion mouse model, while known indirect AMPK activators metformin and canagliflozin were not effective in the same treatment period.^25^

Salsalate is a prodrug of salicylate, which is highly similar to aspirin (acetylsalicylic acid). Salsalate is hydrolyzed to two salicylate molecules in the small intestine. Compared to aspirin, salsalate displays only weak inhibitory effects on cyclo-oxygenases and is associated with minimal bleeding risks and fewer gastro-intestinal side effects.^32^ Salicylate activates AMPK via direct interactions with the Ser108 residue of the AMPK β1-subunit.^33^ These interactions cause allosteric activation and subsequently inhibit dephosphorylation of the activating phosphorylation site, Thr172 of the α-subunit. However, next to its AMPK-activating effects, salicylate can also increase mitochondrial uncoupling and inhibit inflammation and cell proliferation, through multiple distinct mechanisms.

Salsalate is a prime candidate for drug repurposing in ADPKD. In our previous study, the experimental design was to study a treatment effect. Consequently, the observed histopathologic and molecular differences between PKD animals and salsalate-treated animals are largely a reflection of the healthier phenotype, rather than treatment-induced changes. In these kidneys it is virtually impossible to identify molecular mechanisms through which salsalate exerts its beneficial effects. Therefore, in the current study, we aimed to gain more detailed insights into the early and direct effects of salsalate administration on metabolism and signaling, using a new experimental setup, in which mildly cystic PKD animals are treated with salsalate for a short period of time and are compared either to untreated PKD animals with a similar phenotype or with wildtype animals. To this aim, quantitative metabolomic analysis as well as transcriptomic analysis was performed in adult PKD mouse kidneys upon short salsalate treatment.

## Results

### *Pkd1* deletion induces metabolic reprogramming in the kidney

**Figure 1:**
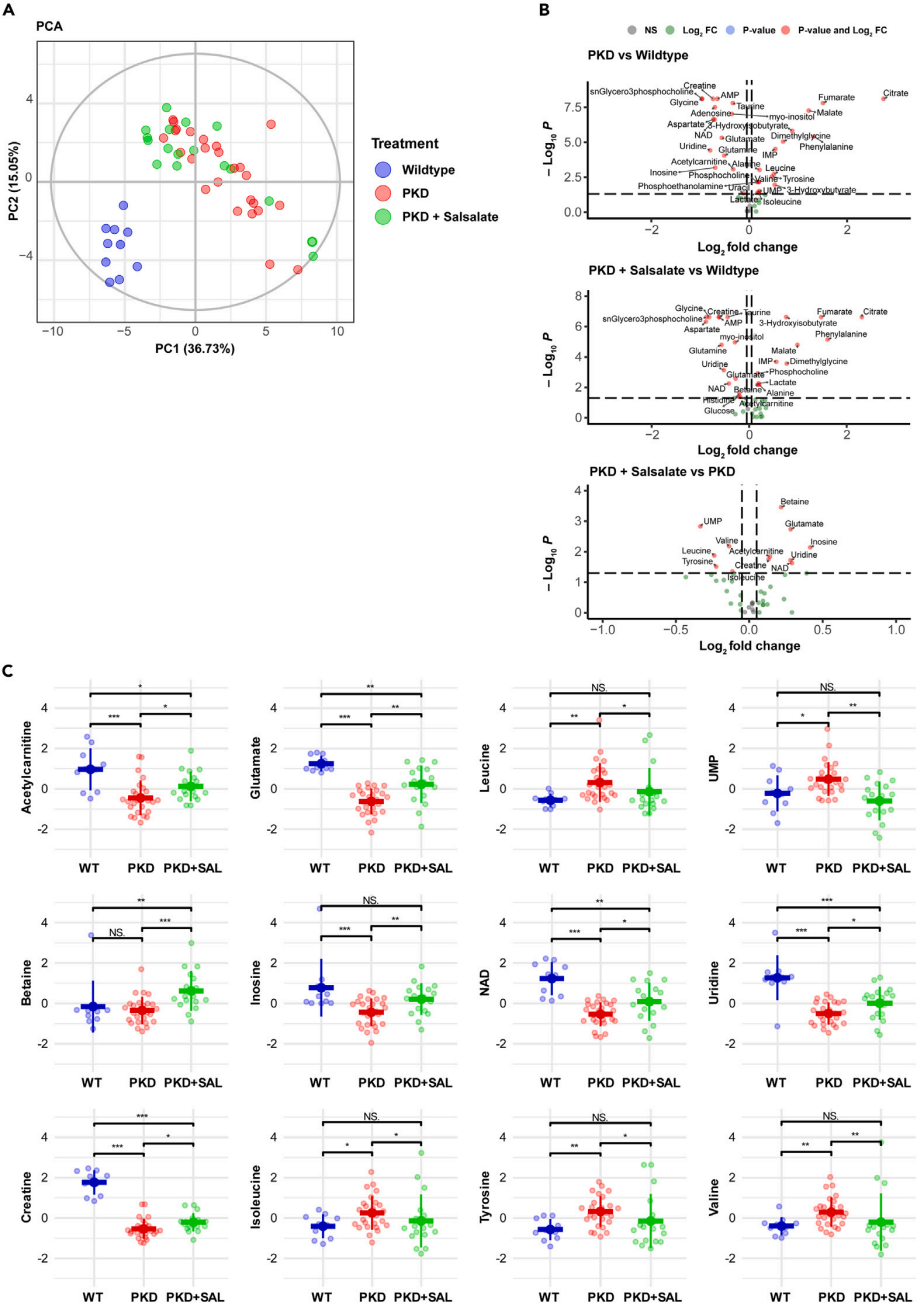
Long-term salsalate treatment attenuates metabolic reprogramming in PKD (A) Principal-component analysis score plot of the different treatment groups. While clear separation between wildtype and PKD animals was observed, salsalate-treated PKD animals were not well distinguishable from untreated PKD animals. Each dot represents an individual animal. The cumulative variance covered by the first two principal components was 51.78%. (B) Volcano plots comparing metabolites between PKD and WT (top), PKD + SAL and WT (middle) and PKD + SAL and PKD (bottom). Annotated metabolites are significantly different between the two groups in the comparison. (C) Scaled metabolite concentrations (μg/mg dry insoluble pellet) of 12 metabolites significantly changed by long-term salsalate treatment. For a majority of the metabolites, salsalate treatment brought metabolite concentrations closer to wildtype levels. Top row, from left to right: acetylcarnitine, glutamate, leucine, UMP. Middle row, from left to right: betaine, inosine, NAD, uridine. Bottom row, from left to right: creatine, isoleucine, tyrosine, valine. Data presented is mean ± SD. Each dot represents an individual mouse kidney. ∗p < 0.05, ∗∗p < 0.01, ∗∗∗p < 0.001, measured by Mann-Whitney U non-parametric univariate test. PC = principal component, WT = wildtype, SAL = salsalate, N.S. = non-significant.

Previously, we have shown that long treatment with the direct AMPK activator salsalate is capable to significantly improve kidney survival in the iKspCre-*Pkd1*^del^ model, compared to untreated *Pkd1* mutant mice.^25^ In addition, we also observed a reduction in 2KW/BW%, cystic index, and blood urea nitrogen (BUN) levels after salsalate treatment. To further investigate the metabolic changes induced by both *Pkd1* gene disruption and a long salsalate treatment, we performed NMR metabolomic analysis to quantitatively measure metabolite levels within cystic kidneys with and without long salsalate treatment. We were able to annotate 43 metabolites; 31 out of these 43 metabolites were significantly different between wildtype and PKD animals (Figure 1B; Table 1; Table S1). Principal-component analysis (PCA) showed a clear separation between wildtype and PKD mice (Figure 1A), with a cumulative variance covered by the first two principal components of 51.78%. We also compared our findings with metabolomic analyses performed by other groups on cystic kidneys of different PKD mouse models (Table S2).^14^^,^^34^^,^^35^ In general, our data are largely in agreement with the other studies, all indicating a distinct metabolic reprogramming in cystic kidneys. However, subtle differences are visible, which are most likely explained by differences in animal model, age, and metabolomic analytical techniques.

**Table 1:** Kidney metabolite concentrations of long treatment salsalate study

Metabolite	Wildtype (±SD)	PKD (±SD)	PKD + Salsalate (±SD)
3-hydroxybutyrate	0,0313 (0,0117)	0,0450 (0,0157)∗	0,0376 (0,0049)
3-hydroxyisobutyrate	0,0120 (0,0016)	0,0220 (0,0046)∗∗∗	0,0203 (0,0027)∗∗∗
AMP	1,8373 (0,1242)	1,1696 (0,2230)∗∗∗	1,1927 (0,1879)∗∗∗
Acetylcarnitine	0,1444 (0,0216)	0,1151 (0,0178)∗∗∗	0,1269 (0,0154)∗#
Adenosine	0,0984 (0,0110)	0,0605 (0,0139)∗∗∗	0,0793 (0,0292)
Alanine	0,3916 (0,0211)	0,4532 (0,0526)∗∗∗	0,4526 (0,0725)∗∗
Asparagine	0,0808 (0,0200)	0,0890 (0,0393)	0,0951 (0,0376)
Aspartate	1,0967 (0,2250)	0,6587 (0,1354)∗∗∗	0,5924 (0,1229)∗∗∗
Betaine	0,5928 (0,1240)	0,5742 (0,0651)	0,6672 (0,0948)∗∗###
Choline	0,1339 (0,0153)	0,1349 (0,0519)	0,1236 (0,0381)
Citrate	0,2876 (0,0464)	1,9247 (1,1474)∗∗∗	1,4265 (0,9523)∗∗∗
Creatine	0,7697 (0,0811)	0,4608 (0,0651)∗∗∗	0,5046 (0,0611)∗∗∗#
Dimethylglycine	0,0054 (0,0014)	0,0087 (0,0018)∗∗∗	0,0092 (0,0026)∗∗∗
Fumarate	0,0145 (0,0055)	0,0411 (0,0168)∗∗∗	0,0402 (0,0124)∗∗∗
Glucose	3,9058 (0,5990)	3,5018 (0,8243)	3,3540 (0,7738)∗
Glutamate	4,1411 (0,2765)	2,8083 (0,4563)∗∗∗	3,4140 (0,6663)∗∗##
Glutamine	1,0684 (0,1268)	0,7499 (0,2287)∗∗∗	0,7212 (0,1368)∗∗∗
Glycine	1,6395 (0,1659)	0,8398 (0,1155)∗∗∗	0,9282 (0,1548)∗∗∗
Histidine	0,1056 (0,0231)	0,0906 (0,0146)	0,0918 (0,0239)∗
Hypoxanthine	0,0744 (0,0247)	0,0730 (0,0181)	0,0863 (0,0210)
IMP	0,1365 (0,0283)	0,1976 (0,0307)∗∗∗	0,2008 (0,0322)∗∗∗
Inosine	0,1341 (0,0605)	0,0827 (0,0282)∗∗∗	0,1103 (0,0333)##
Isoleucine	0,0913 (0,0119)	0,1046 (0,0166)∗	0,0966 (0,0264)#
Lactate	1,9845 (0,2344)	2,2382 (0,3672)∗	2,2726 (0,2181)∗∗
Leucine	0,1523 (0,0233)	0,2160 (0,0690)∗∗	0,1831 (0,0844)#
Malate	0,3069 (0,0639)	0,7125 (0,2685)∗∗∗	0,6094 (0,2747)∗∗∗
Methionine	0,1421 (0,0177)	0,1634 (0,0439)	0,1779 (0,0631)
Myo-inositol	4,6834 (0,3796)	3,6619 (0,4542)∗∗∗	3,8244 (0,3414)∗∗∗
NAD	1,2833 (0,2315)	0,7863 (0,1649)∗∗∗	0,9617 (0,2659)∗∗#
Niacinamide	0,0414 (0,0152)	0,0445 (0,0105)	0,0524 (0,0232)
Phenylalanine	0,0617 (0,0227)	0,1537 (0,0645)∗∗∗	0,1880 (0,1293)∗∗∗
Phosphocholine	1,0364 (0,0583)	1,1542 (0,1484)∗∗	1,1706 (0,1062)∗∗
Phosphoethanolamine	1,2099 (0,0846)	1,3772 (0,1728)∗∗	1,3223 (0,1920)
sn-Glycero-3-phosphocholine	11,0878 (0,9611)	5,6711 (1,4325)∗∗∗	6,0422 (1,3291)∗∗∗
Taurine	8,3204 (0,3478)	6,5938 (0,6781)∗∗∗	6,1229 (0,6739)∗∗∗
Threonine	0,2044 (0,0201)	0,2564 (0,0990)	0,2273 (0,0728)
Tyrosine	0,1425 (0,0321)	0,1963 (0,0450)∗∗	0,1679 (0,0811)#
UDP-N-acetylglucosamine	0,2480 (0,0575)	0,2844 (0,0606)	0,2983 (0,0772)
UMP	0,2549 (0,0504)	0,2948 (0,0459)∗	0,2340 (0,0540)##
Uracil	0,0388 (0,0184)	0,0357 (0,0053)∗	0,0381 (0,0069)
Uridine	0,1968 (0,0524)	0,1131 (0,0244)∗∗∗	0,1374 (0,0385)∗∗∗#
Valine	0,1447 (0,0138)	0,1658 (0,0220)∗∗	0,1507 (0,0435)∗∗

After *Pkd1* deletion on PN18, mice received salsalate treatment from PN40 until 50% of the control group (no treatment) reached ESRD. Kidneys were then bisected and snap-frozen in liquid nitrogen. Metabolites were extracted in methanol (3:1 ratio to KW), after which ^1^H-NMR metabolomics was performed. 43 metabolites were detectable and quantifiable. Data are shown as mean ± SD. ∗p < 0.05, ∗∗p < 0.01, ∗∗∗p < 0.001 vs. wild type. #p < 0.05, ##p < 0.01, ###p < 0.001 vs. PKD, measured by Mann-Whitney U non-parametric univariate test.

### Long-term salsalate treatment attenuates metabolic reprogramming effects in PKD

Next, we focused on the metabolic changes induced by salsalate treatment in PKD. Although the effect of salsalate on the measured metabolites is limited, levels of individual metabolites are significantly different. Compared to PKD animals, we observed a significant change in 12 out of the 43 measured metabolites, most of which are “reverting back” toward their wildtype levels (Figure 1B, Table 1). We saw increased levels of acetylcarnitine, betaine, creatine, glutamate, inosine, NAD^+^, and uridine in salsalate-treated animals, while also seeing decreased levels of isoleucine, leucine, tyrosine, valine, and UMP in salsalate-treated animals (Figure 1C). These 12 metabolites might be an indication toward pathways affected by salsalate treatment in ADPKD. However, an alternative option might be that changes in these metabolites are (in part) a reflection of the more healthier phenotype of salsalate-treated PKD animals, when compared to untreated PKD animals. Concomitantly, the 12 metabolites are largely reflective of the main metabolic processes altered in PKD: fatty acid metabolism (acetylcarnitine), amino acid metabolism in relation to fueling of the tricarboxylic acid (TCA) cycle (creatine, glutamate), ketone body formation (isoleucine, leucine, tyrosine), nucleotide synthesis (inosine, UMP, uridine), and oxidative phosphorylation (NAD^+^). This is supported by our previously published transcriptome data, which show a significant attenuated expression of genes involved in these metabolic pathways, as well as genes involved in inflammation and fibrosis, upon long-term salsalate treatment.^25^

### Mild cystic mice show early signs of metabolic reprogramming

**Figure 2:**
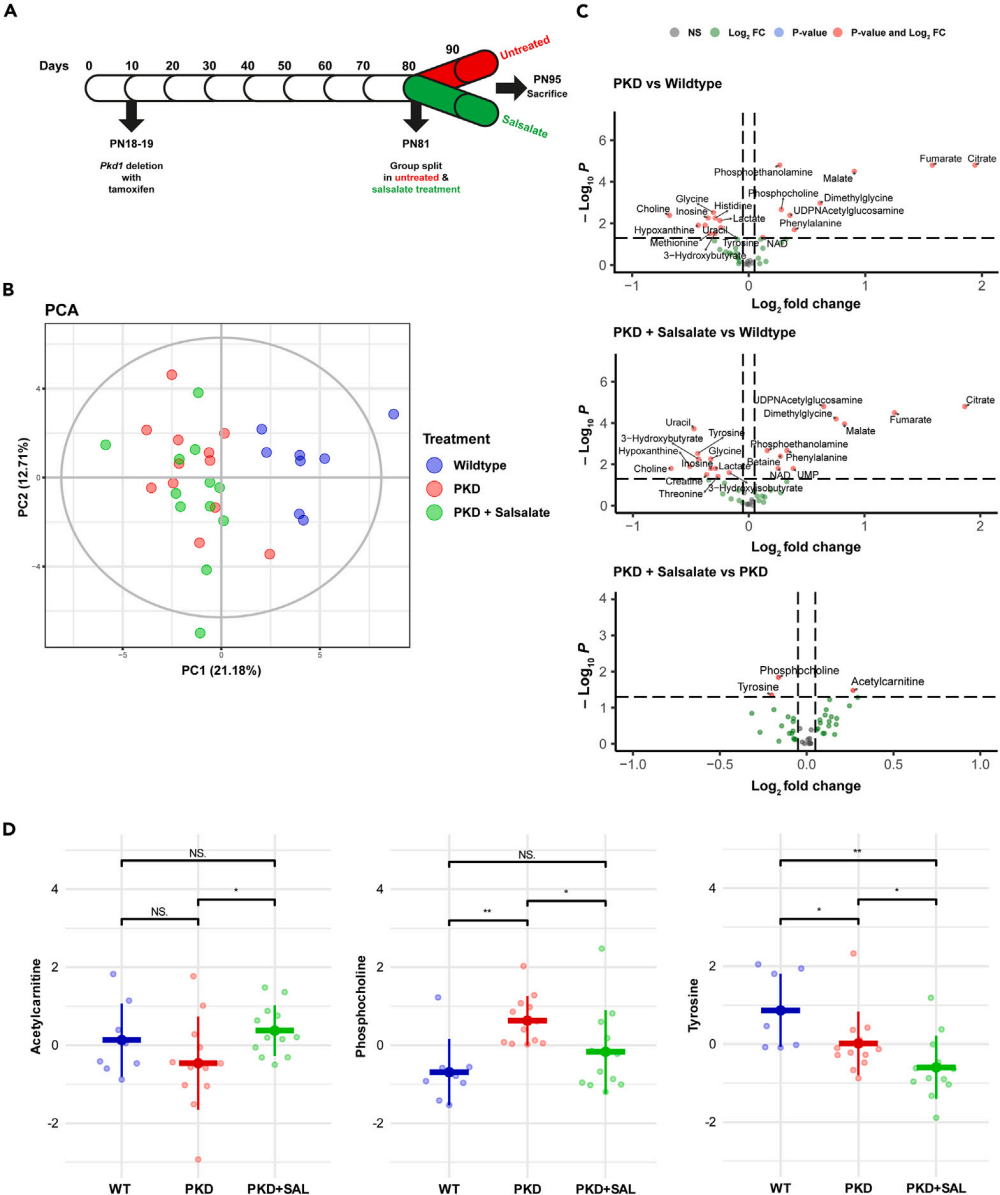
Short-term salsalate treatment has small effects on the PKD metabolome (A) Mouse experimental timeline. iKspCre-*Pkd1*^del^ mice were treated with 150 mg/kg tamoxifen to induce *Pkd1* deletion at PN18/19. At PN81, mice were split in two groups, 1 group remained untreated for the last two weeks, while the other group received salsalate treatment (400 mg/kg/day in diet) for two weeks. At PN95, all mice were sacrificed. (B) Principal-component analysis score plot of the different treatment groups. While clear separation between wildtype and PKD animals was observed, salsalate-treated PKD animals were undistinguishable from PKD animals. Each dot represents an individual animal. The cumulative variance covered by the first two principal components was 33.89%. (C) Volcano plots comparing metabolites between PKD and WT (top), PKD + SAL and WT (middle) and PKD + SAL and PKD (bottom). Annotated metabolites are significantly different between the two groups in the comparison. (D) Scaled metabolite concentrations (μg/mg dry insoluble pellet) of 3 metabolites changed by short-term salsalate treatment. Acetylcarnitine & phosphocholine revert back to wildtype levels, while tyrosine does not. Data presented are mean ± SD. Each dot represents a mouse kidney. ∗p < 0.05, ∗∗p < 0.01, measured by Mann-Whitney U non-parametric univariate test. PN = post-natal, PC = principal component, WT = wildtype, SAL = salsalate, N.S. = non-significant.

Considering the option that the observed differences between PKD animals and salsalate-treated animals are largely a reflection of the healthier phenotype, rather than treatment-induced changes, we set out a second *in vivo* experiment in which PKD mice were treated with salsalate for only a short period of time. This setup (Figure 2A) enables us to better study the molecular effects induced by salsalate, without having phenotypic interference. We induced *Pkd1* gene deletion with tamoxifen at post-natal day (PN) 18–19. At PN81, mice were split into two groups: one group remained without treatment until sacrifice at PN95, while the other group received salsalate treatment (400 mg/kg/day) until sacrifice at PN95. In order to prevent as much phenotypic bias as possible, we selected, from both groups, twelve mice (out of 36) with a similarly mild cystic phenotype (2KW/BW 2.5–4, Table S3) for further analyses. A PCA plot shows clear separation between wildtype and PKD animals (Figure 2B), with a cumulative variance covered by the first two principal components of 33.89%. Furthermore, a univariate analysis showed that 19 metabolites out of 44 detectable metabolites were significantly different between wildtype and PKD animals (Figure 2C, Table 2; Table S4). Comparison of both studies (long treatment vs. short treatment) for the metabolic alterations between wildtype and PKD showed a limited overlap (i.e., increased levels of citrate, dimethylglycine, fumarate, malate, phenylalanine, phosphocholine & phosphoethanolamine; decreased levels of inosine, glycine & uracil), but, in general, there is disconnect with some unaltered metabolites, or metabolites showing an opposite pattern. This is likely explained by the differences in the PKD phenotypes of both studies, which are reflected by their 2KW/BW% (Table S3). These data suggest that the differences in metabolites we observe are likely due to the more advanced disease condition of the PKD mice in the long treatment study, causing secondary changes after the initial changes we observe in the mild disease condition.

**Table 2:** Kidney metabolite concentrations of short treatment salsalate study

Metabolite	Wildtype (±SD)	PKD (±SD)	PKD + Salsalate (±SD)
3-hydroxybutyrate	0,0350 (0,0051)	0,0286 (0.0060)∗	0,0259 (0.0073)∗∗
3-hydroxyisobutyrate	0,0100 (0,0014)	0,0099 (0.0032)	0,0089 (0.0013)∗
Acetylcarnitine	0,0959 (0,0192)	0,0837 (0.0244)	0,1008 (0.0133)#
AMP	1,1135 (0,1062)	1,1232 (0.1194)	1,1403 (0.1011)
Adenosine	0,0660 (0,0104)	0,0619 (0.0074)	0,0653 (0.0133)
Alanine	0,3125 (0,0359)	0,3400 (0.0302)	0,3440 (0.0703)
Asparagine	0,0524 (0,0068)	0,0654 (0.0174)	0,0543 (0.0211)
Aspartate	0,6435 (0,1667)	0,5741 (0.0923)	0,5789 (0.0840)
Betaine	0,5111 (0,2806)	0,5490 (0.1055)	0,6176 (0.1144)∗∗
Choline	0,2811 (0,0801)	0,1756 (0.0735)∗∗	0,1765 (0.0870)∗
Citrate	0,1265 (0,0408)	0,4854 (0.1401)∗∗∗	0,4609 (0.1701)∗∗∗
Creatine	0,4999 (0,1229)	0,4079 (0.0823)	0,3885 (0.0582)∗
Dimethylglycine	0,0028 (0,0005)	0,0042 (0.0018)∗∗	0,0047 (0.0016)∗∗∗
Fumarate	0,0099 (0,0020)	0,0295 (0.0081)∗∗∗	0,0237 (0.0069)∗∗∗
Glucose	2,1753 (0,5643)	2,0502 (0.3447)	2,3118 (0.5020)
Glutamate	2,7534 (0,4267)	2,5559 (0.3078)	2,7637 (0.3900)
Glutamine	0,5427 (0,1246)	0,5796 (0.0521)	0,6186 (0.1566)
Glycine	1,2133 (0,1741)	0,9836 (0.1052)∗∗	0,9664 (0.1478)∗∗
Histidine	0,0648 (0,0090)	0,0531 (0.0062)∗∗	0,0560 (0.0127)
Hypoxanthine	0,1372 (0,0458)	0,1017 (0.0171)∗	0,0964 (0.0182)∗
IMP	0,1810 (0,0297)	0,1916 (0.0222)	0,2273 (0.0793)
Inosine	0,1745 (0,0316)	0,1371 (0.0273)∗∗	0,1391 (0.0290)∗
Isoleucine	0,0646 (0,0131)	0,0583 (0.0060)	0,0610 (0.0067)
Lactate	2,2616 (0,3894)	1,9047 (0.1372)∗∗	1,8542 (0.2573)∗
Leucine	0,1317 (0,0381)	0,1109 (0.0093)	0,1127 (0.0191)
Malate	0,1721 (0,0415)	0,3224 (0.0500)∗∗∗	0,3053 (0.0655)∗∗∗
Methionine	0,0816 (0,0174)	0,0648 (0.0095)∗	0,0642 (0.0129)
Myo-inositol	3,2799 (0,1769)	3,0676 (0.2129)	3,1918 (0.2917)
NAD	1,1642 (0,0920)	1,2663 (0.1023)∗	1,3881 (0.2267)∗
Niacinamide	0,0338 (0,0117)	0,0346 (0.0103)	0,0326 (0.0115)
Phenylalanine	0,0656 (0,0091)	0,0860 (0.0178)∗	0,0824 (0.0095)∗∗
Phosphocholine	0,7226 (0,0995)	0,8771 (0.0742)∗∗	0,7839 (0.1248)#
Phosphoethanolamine	0,4589 (0,0311)	0,5518 (0.0481)∗∗∗	0,5124 (0.0719)∗∗
Succinate	0,5333 (0,0463)	0,5329 (0.0433)	0,5427 (0.0758)
sn-Glycero-3-phosphocholine	12,1109 (2,4684)	11,3333 (1.4083)	12,4028 (2.1135)
Taurine	5,1472 (0,3412)	5,1014 (0.3288)	4,8403 (0.4815)
Threonine	0,2178 (0,0584)	0,2063 (0.0386)	0,1810 (0.0229)∗
Tyrosine	0,1163 (0,0197)	0,0985 (0.0172)∗	0,0856 (0.0170)∗∗#
UDP-N-acetylglucosamine	0,1719 (0,0436)	0,2196 (0.0463)∗∗	0,2693 (0.0442)∗∗∗
UDP-glucose	0,2510 (0,0989)	0,2780 (0.0610)	0,3038 (0.0503)
UMP	0,1270 (0,0238)	0,1544 (0.0330)	0,1657 (0.0367)∗
Uracil	0,0459 (0,0096)	0,0355 (0.0064)∗	0,0331 (0.0054)∗∗∗
Uridine	0,1380 (0,0216)	0,1277 (0.0110)	0,1374 (0.0187)
Valine	0,1012 (0,0135)	0,0988 (0.0089)	0,1105 (0.0289)

After *Pkd1* deletion on PN18, mice remained untreated until PN81, at which point half of the mice received salsalate treatment for 14 days, and the other half remained untreated. Kidneys were then bisected and snap-frozen in liquid nitrogen. Metabolites were extracted in methanol (3:1 ratio to KW), after which ^1^H-NMR metabolomics was performed. 44 metabolites were detectable and quantifiable. Data are shown as mean ± SD. ∗p < 0.05, ∗∗p < 0.01, ∗∗∗p < 0.001 vs. wild type. #p < 0.05, ##p < 0.01, ###p < 0.001 vs. PKD, measured by Mann-Whitney U non-parametric univariate test.

### Short salsalate treatment induces small changes in the PKD metabolome

We then focused on changes induced by short salsalate treatment in PKD. Due to the short duration of treatment, no large differences were expected, and changes should likely be attributed to the molecular working mechanisms of salsalate. PCA analysis (Figure 2B) shows indeed no clear clustering of the PKD and PKD + salsalate groups, which is reflecting the similar disease phenotypes in both groups. We observed three metabolites that are significantly altered upon short salsalate treatment: acetylcarnitine, phosphocholine, and tyrosine (Figures 2C and 2D). Acetylcarnitine and phosphocholine are reverting back to wildtype levels (Figure 2D) upon salsalate treatment, suggesting that these two metabolites are close to or involved in the molecular working mechanisms of salsalate. Acetylcarnitine is the most common derivative of carnitine, a well-known regulator of fatty acid transportation and oxidation. Phosphocholine is an intermediate molecule in the synthesis pathway of phosphatidylcholine, which is the most abundant phospholipid in mammalian cells. Interestingly, in PKD kidneys, tyrosine levels are lower compared to wild types, and salsalate treatment caused an even further reduction, suggesting a non-PKD relevant effect of salsalate on tyrosine. Of these three metabolites, acetylcarnitine (and tyrosine) also changed in the long salsalate treatment, further strengthening the role of acetylcarnitine in the molecular mechanism of salsalate.

### Metabolite-metabolite correlations show salsalate treatment associates with altered purine metabolism

**Figure 3:**
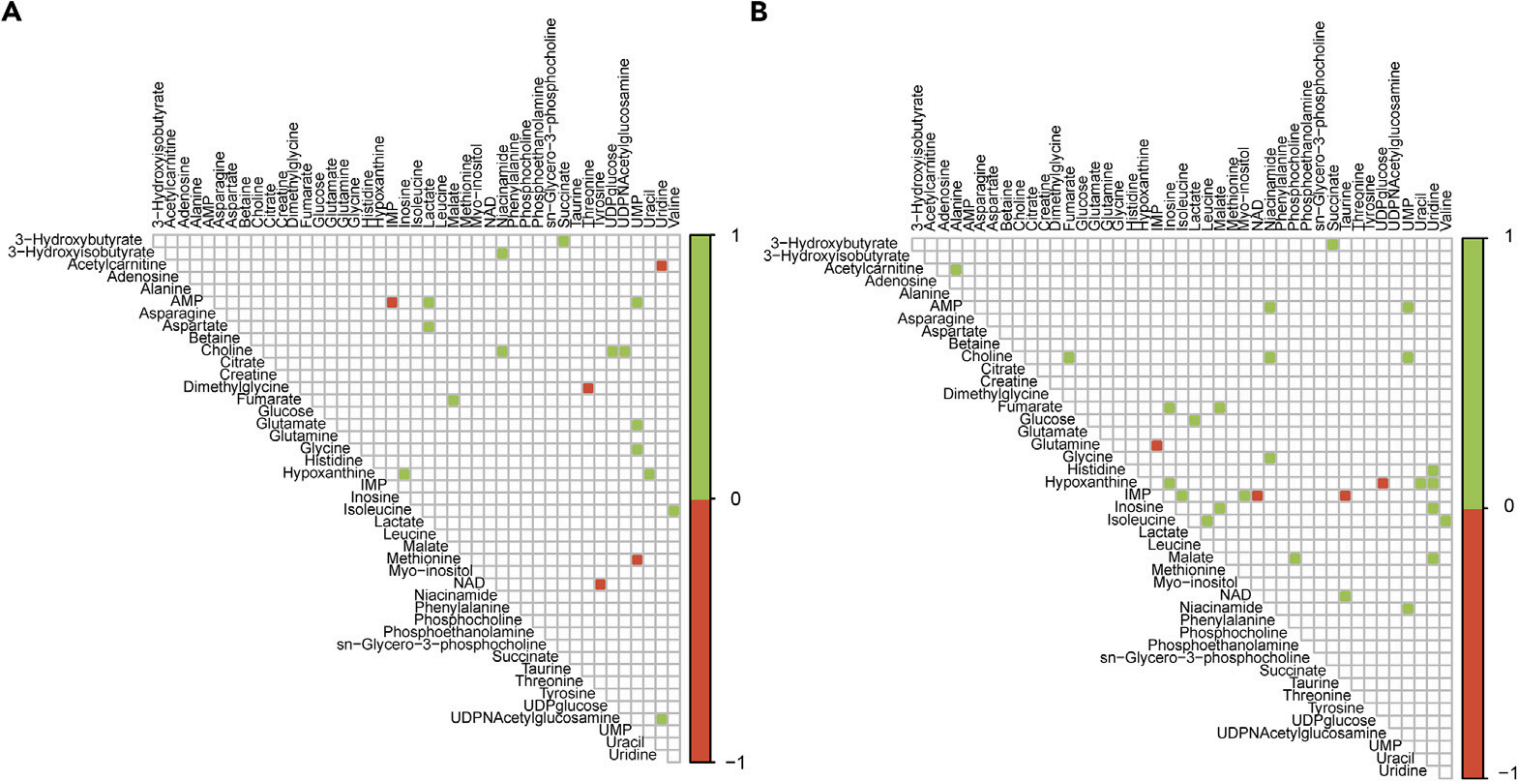
Metabolite-metabolite correlations between wildtype, PKD and PKD + SAL animals (A) Correlation matrix between metabolite concentrations measured in PKD vs. WT animals. A positive correlation (green) indicates both metabolites in the correlation show the same direction change between PKD and WT animals, whereas that a negative correlation (red) indicates a change in the opposite direction. (B) Correlation matrix between metabolite concentrations measured in PKD + SAL vs. PKD animals. Values −1 and +1 are arbitrary and are a way to visually indicate whether there is a correlation in the same (green) or in the opposite direction (red). The actual Pearson’s correlation coefficients and p values from each metabolite-metabolite pairs per experimental group (wild-type, PKD, and PKD+SAL) can be found in Tables S5, S6, and S7.

**Figure 4:**
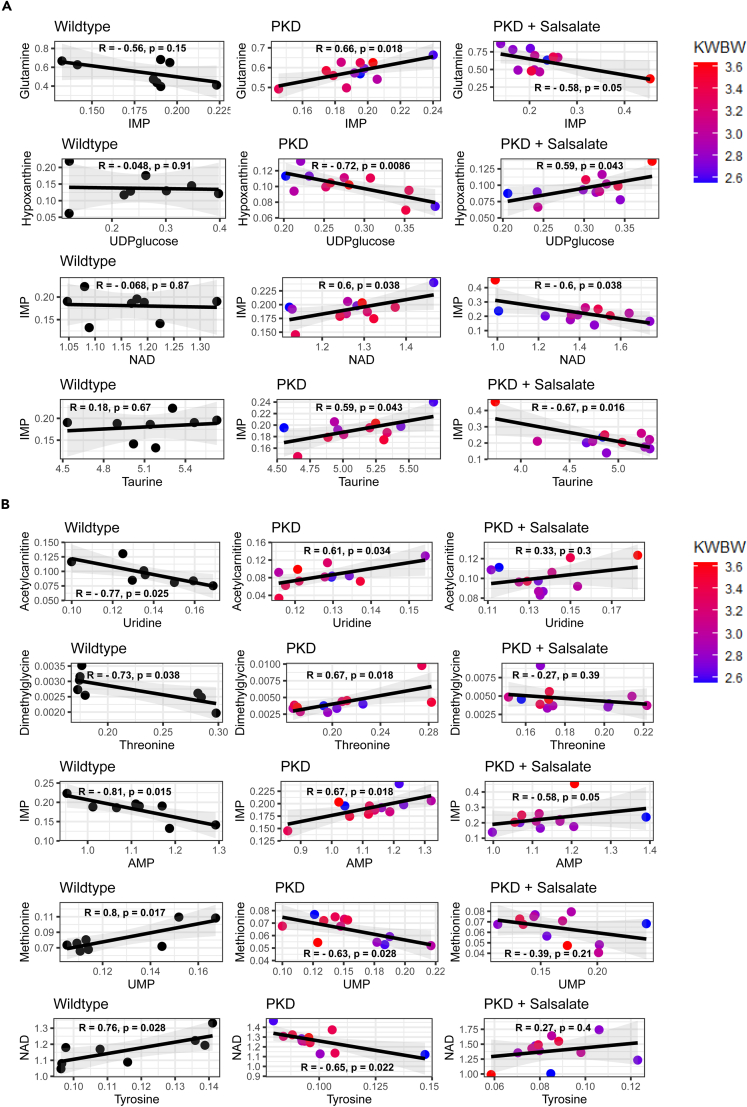
Metabolite-metabolite correlation associate salsalate treatment with changes in purine metabolism (A) Metabolite-metabolite correlation pairs with an opposite correlation pattern between PKD + SAL vs. PKD. From top to bottom: glutamine-IMP, hypoxanthine-UDP-glucose, IMP-NAD & IMP-taurine. Color shading refers to cystic phenotype (2KW/BW) of the mouse; the numerical value refers to the severity of the cystic phenotype, with low ratios meaning a mild phenotype, and higher values meaning a more severe phenotype. (B) Metabolite-metabolite correlation pairs showing metabolites with an opposite correlation pattern between PKD vs. WT, which is corrected toward WT by salsalate treatment. From top to bottom: acetylcarnitine-uridine, dimethylglycine-threonine, IMP-AMP, methionine-UMP & NAD-tyrosine. Color shading refers to cystic phenotype (2KW/BW) of the mouse. Each dot represents a mouse kidney. R = Pearson’s correlation coefficient, P = p value.

Having identified key metabolites affected by short salsalate treatment, we tried to underpin their functional role. We reasoned that building a metabolic network on a handful of metabolites comes with a risk of obtaining far too general and poorly interpretable output. As an alternative we focused on correlations between the individual metabolites (Tables S5, S6, and S7). We hypothesized that, if the concentrations of two metabolites are correlated, it suggests that they are produced or consumed by an interconnected set of enzymes. Consequently, by exploring such correlations it would be possible to get an indication on how functional relationships between the metabolites change depending on experimental condition (e.g., wildtype, PKD, PKD + Salsalate). Thus, Figure 3 shows correlations among individual metabolites between wildtype and PKD mice (Figure 3A), and between PKD and PKD + Salsalate mice (Figure 3B). First, we zoomed in on metabolite-metabolite correlations that show an opposite correlation between PKD + Salsalate and PKD (i.e., metabolite pairs have a positive correlation in PKD, and a negative correlation in PKD + SAL, or vice versa). We found four metabolite pairs with this pattern, namely glutamine-IMP, hypoxanthine-UDP-glucose, IMP-NAD, and IMP-taurine (Figure 4A). All four pairs are absent in the wildtype-PKD correlation matrix, indicating that potential effects are salsalate specific, and not related to the PKD phenotype. Next, we zoomed in on metabolite-metabolite correlations which show a negative correlation between wild type and PKD and were also “corrected” by salsalate treatment (i.e., metabolite pairs have an opposite correlation in wildtype and PKD, while PKD + Salsalate shows a corrected pattern toward wild type). This revealed another five metabolite pairs which are likely influenced by salsalate treatment, namely acetylcarnitine-uridine, dimethylglycine-threonine, IMP-AMP, methionine-UMP, and NAD-tyrosine (Figure 4B). With 3 out of 5 metabolite pairs containing a purine (acetylcarnitine-uridine, IMP-AMP, and methionine-UMP), this strengthens the suggestion that salsalate treatment is affecting purine metabolism in PKD.

### Altered AMPK signaling upon short salsalate treatment in PKD animals

**Figure 5:**
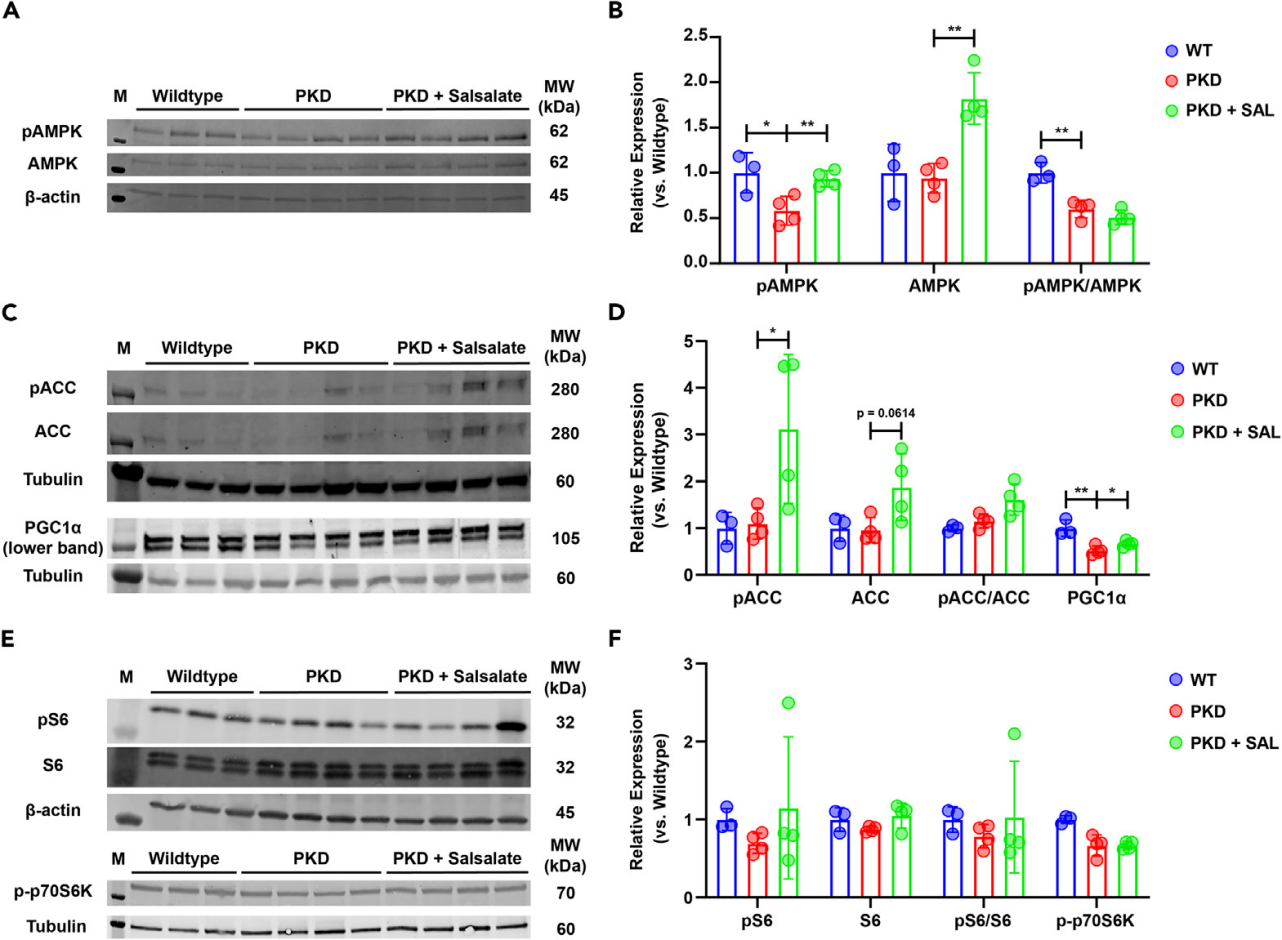
AMPK signaling upon short salsalate treatment in PKD animals (A) Western blotting for pAMPK and AMPK on protein extracts isolated from wildtype, PKD and PKD + SAL kidneys. β-actin protein expression was used as an internal loading control. (B) Quantification of the pAMPK and AMPK blots shown in (A), relative to β-actin. Both pAMPK and AMPK expression is increased in PKD + SAL animals, compared to PKD, resulting in a non-significant difference in the pAMPK/AMPK ratio. (C) Western blotting for pACC, ACC, and PGC1α on protein extracts isolated from wildtype, PKD, and PKD + SAL kidneys. Tubulin protein expression was used as an internal loading control. Please note that for the PGC1α blot, only the lower band visible corresponds to the PGC1α protein. (D) Quantification of the pACC, ACC, and PGC1α blots shown in (C), relative to tubulin. pACC expression is increased significantly in PKD + SAL animals, compared to PKD, while ACC expression is trending toward a significant increase (p = 0.0614). This results in a non-significant difference in the pACC/ACC ratio. PGC1α expression is reduced in PKD animals, which is attenuated by short salsalate treatment. (E) Western blotting for pS6, S6, and p-p70S6K on protein extracts isolated from wildtype, PKD, and PKD + SAL kidneys. β-actin and tubulin protein expression were used as an internal loading control. (F) Quantification of the pS6, S6, and p-p70S6K blots shown in (E), relative to β-actin (pS6, S6) and tubulin (p-p70S6K). No significant differences were detected between wildtype, PKD, and PKD + SAL animals. Each dot represents a mouse kidney (n = 3–4 animals per group). Data presented are mean ± SD. ∗p < 0.05, ∗∗p < 0.01, measured by two-way unpaired Student’s t tests. M = marker, MW = molecular weight, WT = wildtype, SAL = Salsalate, kDa = kilodalton.

Salsalate, upon hydrolysis to salicylate *in vivo*, activates AMPK and its downstream targets by preventing AMPK dephosphorylation of Thr172.^33^ We therefore verified whether a short salsalate treatment could also prevent this dephosphorylation. We found that AMPK phosphorylation at Thr172 is significantly increased in salsalate-treated animals, compared to PKD animals (Figures 5A and 5B). However, this increase was accompanied with an increased total AMPK protein level in salsalate-treated animals (Figures 5A and 5B), resulting in an unchanged pAMPK/AMPK ratio (Figure 5B). We also looked at several downstream targets of AMPK, to further verify salsalate efficacy. Salsalate treatment induced a significant increase in phosphorylation of acetyl-coenzyme A (CoA) carboxylase (ACC) at Ser79, but this was accompanied by an increase in total ACC levels (trending toward significance) (Figures 5C and 5D), resulting in an unchanged pACC/ACC ratio (Figure 5D). Furthermore, short salsalate treatment increased the expression of the mitochondrial biogenesis regulator peroxisome proliferator-activated receptor γ coactivator 1α (PGC1α, Figures 5C and 5D). Activated AMPK can also inhibit the mammalian target of rapamycin (mTOR) pathway, which is an important driver of cyst growth when active. No salsalate-induced changes in phosphorylation of the two mTOR pathway effector proteins, pS6 and p-p70S6K, were observed (Figures 5E and 5F).

### RNA sequencing reveals anti-proliferative and anti-inflammatory effects of salsalate in ADPKD

**Figure 6:**
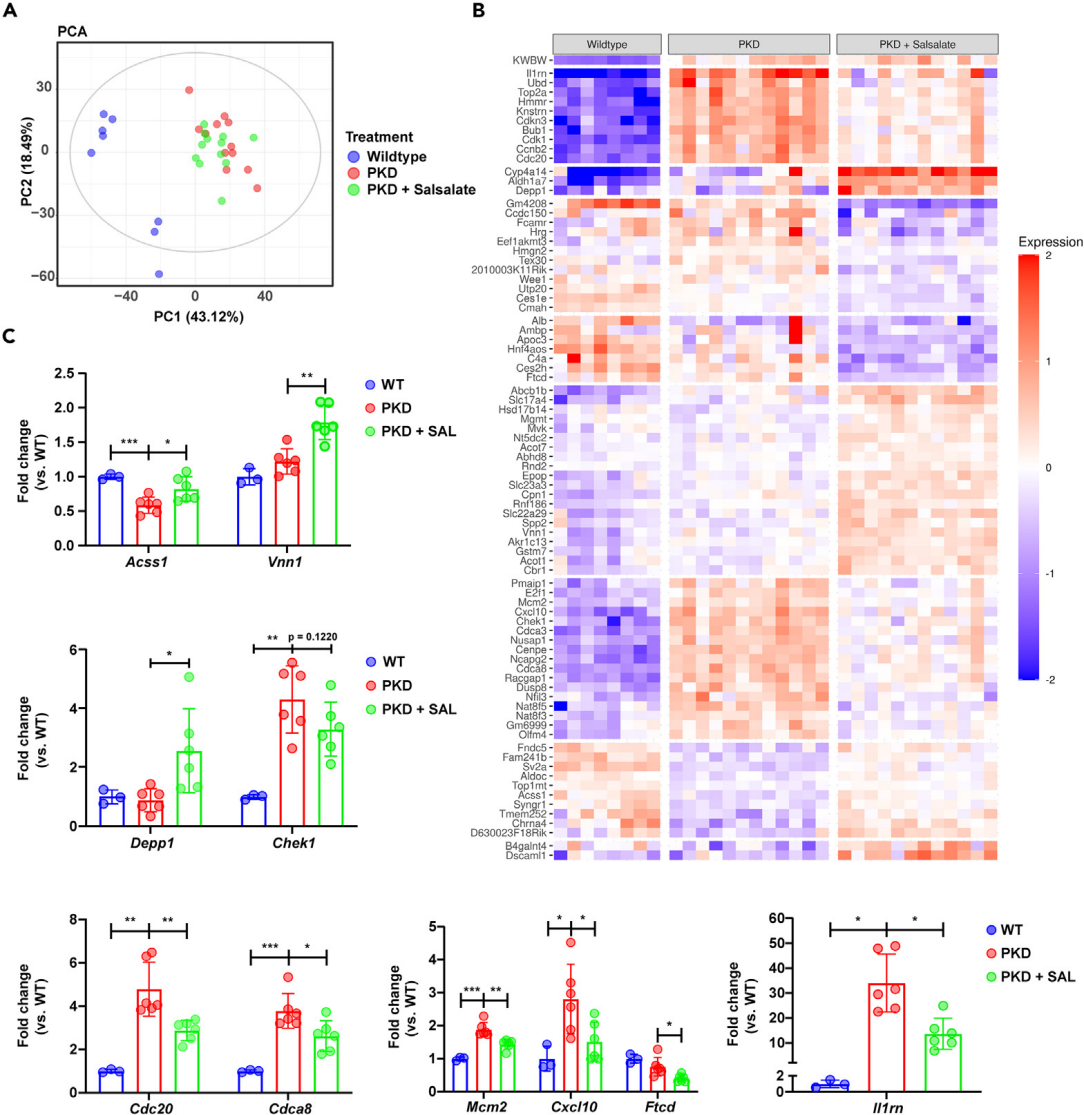
RNA sequencing reveals an anti-proliferative effect of short salsalate treatment (A) Principal-component analysis score plot of the different treatment groups. While clear separation between WT and PKD animals was observed, PKD + SAL animals were undistinguishable from PKD animals. Each dot represents an individual animal. The cumulative variance covered by the first two principal components was 61.61%. (B) Heatmap of the 81 differentially expressed genes between PKD + SAL vs. PKD animals. Genes with a p value (FDR) p < 0.05 were considered significant and were grouped together via unbiased clustering; each column represents a mouse kidney. Red indicates gene expression higher than the average for this gene; blue indicates gene expression lower than the average for this gene. The majority of genes affected, based on RNA sequencing, are involved in cell proliferation, energy metabolism and inflammation. (C) Gene expression of RNA sequencing hit genes in wildtype, PKD, and PKD + SAL animals. Each dot represents a mouse kidney (n = 3–6 animals per group). Data presented are mean ± SD. ∗p < 0.05, ∗∗p < 0.01, ∗∗∗p < 0.001, measured by two-way unpaired Student’s t tests. PC = principal component, WT = wildtype.

To find out more about the salsalate-induced alterations in the transcriptome, we performed RNA sequencing on the samples from the short treatment study. The same 32 (8 wildtypes, 12 PKD, 12 PKD + Salsalate) mice used for NMR metabolomics were used for this. PCA analysis revealed a clear separation between wildtype and PKD animals, while PKD and PKD + Salsalate mice could not be distinguished clearly from each other (Figure 6A). The cumulative variance covered by the first two principal components was 61.61%. Our data reveal 81 significantly differentially expressed genes between PKD and PKD + Salsalate animals (Figure 6B, 35 upregulated and 46 downregulated). 58 out of the 81 genes (72%) were also significantly differentially expressed between wildtype and PKD animals, the majority of which (74%) were also corrected by salsalate treatment back in the direction of wild-type levels. From the differentially expressed gene list, three major themes could be dissected: cell proliferation (27%), energy metabolism (26%), and inflammation (11%). Unbiased clustering of the 81 differentially expressed genes indicates that the salsalate-affected cluster with the largest difference between wildtype and PKD animals contains strongly upregulated genes involved in cell proliferation, such as *Top2a, Cdkn3, Cdk1, Ccnb2 and Cdc20, Mcm2*, and *Cdca8*. (Figure 6B). Moderately downregulated genes corrected by salsalate treatment are to a large extend involved in energy metabolism, such as *Ces1e*, *Depp1*, *Fndc5*, *Aldoc, Vnn1*, *Acss1*, and *Chrna4*. As the number of differentially expressed genes between the PKD + Salsalate and PKD groups is low, differentially expressed genes (false discovery rate [FDR] <0.05, 81 genes), as well as genes with a Log2FC < 0.5/> 0.5 (103 genes), were selected for gene set enrichment analysis (GSEA). The combined group of 184 genes could be split into 55 upregulated genes and 129 downregulated genes. As expected, GSEA revealed only a limited number of significant pathways affected by salsalate treatment but clearly confirms the importance of cell proliferation, inflammation (downregulated), and energy metabolism (upregulated) among the effects of salsalate treatment (Tables S8 and S9). We validated multiple of the RNA sequencing hits with qPCR and confirmed that genes involved in energy metabolism and, more specifically, fatty acid oxidation (FAO) (*Acss1, Vnn1, Depp1*) are upregulated, while genes involved in proliferation (*Chek1, Cdc20, Cdca8, Mcm2*) and inflammation (*Cxcl10, Il1rn, Ftcd*) are downregulated by salsalate treatment (Figure 6C). At the protein level, expression of CDK1 and HMGN2 was not changed by salsalate treatment (Figure S1).

**Figure 7:**
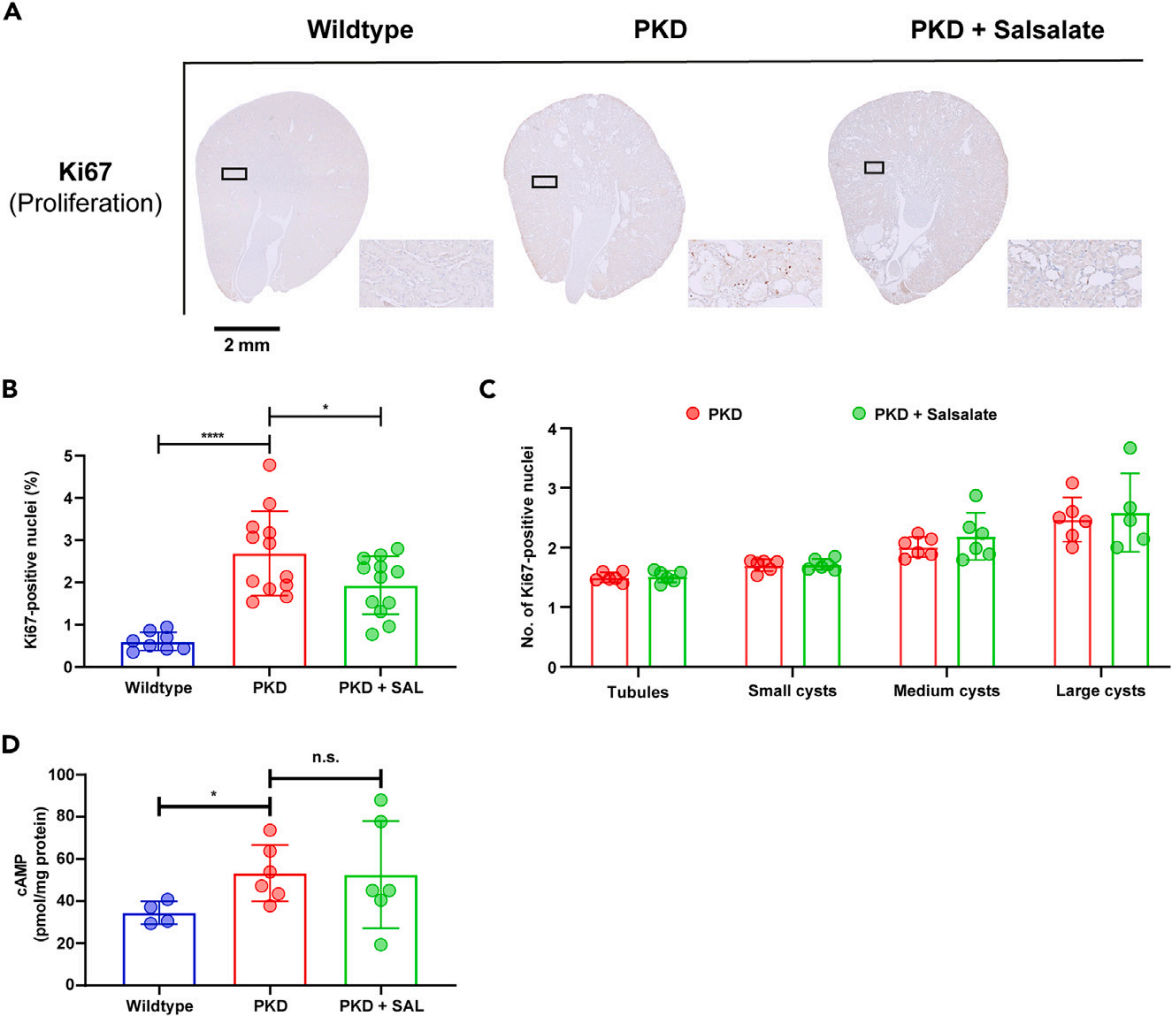
Short salsalate treatment has anti-proliferative effects in PKD (A) Immunohistochemical staining for the proliferation marker Ki67, showing that PKD + Salsalate mice have reduced proliferation compared to PKD mice. (B) Quantification of Ki67^+^ nuclei. PKD animals have significantly more proliferating nuclei (measured by % Ki67^+^ nuclei) compared to wildtype animals, while PKD + Salsalate mice have less proliferating nuclei compared to PKD mice. n = 8–12 animals per group. (C) Quantification of number of Ki67^+^ nuclei in tubules and cysts of different size. Tubules and cyst were manually judged, the tubular/cystic diameter was measured and then split in four groups: The cystic/tubular diameter was measured in pixels and divided in four groups: tubules, small cysts (<1.75x average tubular diameter), medium cysts (1.75-3x average tubular diameter) and large cysts (>3x tubular diameter). n = 6 animals per group. (D) Analysis of kidney cAMP concentrations (pmol/mg protein) show an increased cAMP level in PKD mice compared to WT, while PKD + Salsalate mice do not differ from PKD mice. n = 4–6 animals per group. Data presented are mean ± SD. Each dot represents a mouse kidney. Scalebar = 2 mm ∗p < 0.05, ∗∗∗∗p < 0.0001, measured by two-way unpaired Student’s t tests (D). WT = wildtype, SAL = salsalate.

As increased cell proliferation is an important hallmark in ADPKD pathogenesis, we further focused on the anti-proliferative effects of short salsalate treatment by performing immunohistochemistry for the proliferation marker Ki67. The percentage Ki67-positive nuclei of the total nuclei count was increased in PKD animals compared to wild types and reduced in PKD + Salsalate animals compared to PKD animals (Figures 7A and 7B). Comparing the number of Ki67-positive nuclei in tubules, small-sized, medium-sized, and large-sized cysts, did not show a difference between PKD + Salsalate and PKD animals, (Figure 7C). Because of the importance of elevated cyclic AMP (cAMP) levels in the increased proliferation levels observed in PKD,^36^^,^^37^^,^^38^ we measured cAMP levels in wild-type, PKD, and PKD + Salsalate animals. As expected, we found significantly increased cAMP levels in PKD mice, compared to wild-type mice (Figure 7D). However, short salsalate treatment had no effect on cAMP levels in the kidney.

Although we observed an effect of salsalate on the expression of inflammation genes, B and T cell infiltration at this mild stage of PKD is very limited, and virtually no B and T cell presence could be detected (Figure S2). We also studied macrophage infiltration by staining for the macrophage marker F4/80, which shows higher expression in PKD mice compared to wildtypes (Figure S3). However, no significant effect of the short salsalate treatment was found, although a non-significant trend is visible. Overall, our data indicate that, in addition to altering energy metabolism, salsalate also has an anti-proliferative and anti-inflammatory effect that affects proliferation and inflammation in mild cystic kidneys.

### Combined metabolomic and transcriptomic network analysis confirms the effects of short salsalate treatment on energy metabolism in PKD

**Figure 8:**
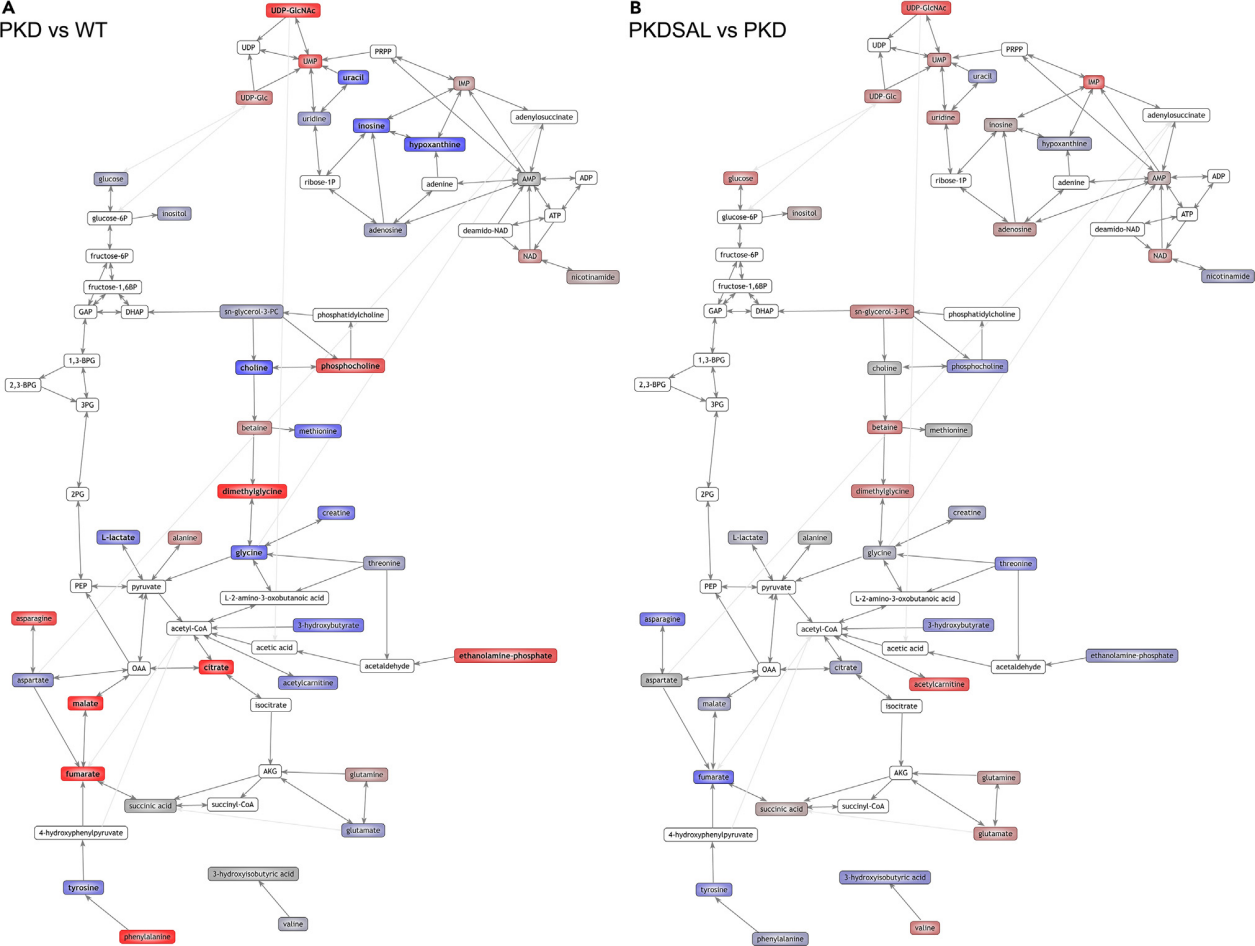
Visual map of metabolic changes induced by *Pkd1* deletion or salsalate treatment (A) Visual representation of metabolic changes, comparing PKD animals versus wildtype animals. Red shaded metabolites are increased in PKD animals, and blue shaded metabolites are decreased in PKD animals. Color intensity reflects the degree of change. Metabolites in bold are significantly different. (B) Visual representation of metabolic changes, comparing PKD + SAL animals versus PKD animals. Red shaded metabolites are increased in PKD + SAL animals, and blue shaded metabolites are decreased in PKD + SAL animals. Color intensity reflects the degree of change. Metabolites in bold are significantly different. Maps were created with the MRN pathway browser tool (see STAR Methods).

In order to gain a better understanding of the system-wide effects of short salsalate treatment on the metabolic pathways, we made use of the Mouse Reaction Network (MRN) to project our metabolomic data onto a proper biochemical network. The comparison between mildly cystic PKD mice and wildtypes confirms our findings and those of others,^14^^,^^34^^,^^35^ which show, next to many other alterations, part of the metabolic reprogramming and accumulation of citrate, fumarate, and malate, confirming TCA cycle dysregulation, a characteristic of Warburg-like metabolic reprogramming (Figure 8A). When comparing PKD + Salsalate and PKD, multiple metabolites are changing back toward wild-type concentrations, but the detected differences are much milder, reflecting the small effects that salsalate has upon short salsalate treatment (Figure 8B). Next, we added our transcriptomic data to the MRN as well, giving us the visualization of a fully integrated biochemical network with both genes and metabolites affected by short salsalate treatment in PKD (Figures S4–S7). Specifically, we again observe that salsalate treatment positively affects FAO, by increasing acetylcarnitine concentrations, as well as modulation of the expression of pathway-related genes *Acacb, Acss1, Aldh1a3, Crat, Fasn, Lpcat2*, and *Mcat* (Figure S5A). Together with the mild observed changes in TCA cycle metabolites citrate, fumarate, and malate, and the modulation of TCA-cycle-associated genes (Figure S5B), this re-confirms salsalate’s effects on reverting the Warburg-like metabolic reprogramming observed in ADPKD. In addition, metabolites and genes involved in choline metabolism also show small salsalate-induced changes, confirming our findings at metabolite level (Figure S6). Moreover, the network analysis also shows various small changes in various purine molecules, such as hypoxanthine, IMP, UMP, and uracil, as well as genes involved in purine interconversions, which is in line with our metabolite-metabolite correlation analyses (Figure S7).

## Discussion

Currently, the vasopressin V2 receptor antagonist tolvaptan is the only approved drug available to ADPKD patients, but it has side effects and can only be prescribed to a limited patient subset. Therefore, patients remain in need of alternatives which are both safe and effective. We have shown that the AMPK activator salsalate reduces kidney cyst growth in a clinically relevant ADPKD *in vivo* model.^25^^,^^39^ However, due to the chosen experimental design, which contains inherent phenotypic bias, it is impossible to identify molecular mechanisms through which salsalate exerts its beneficial effects. Therefore, we here present a new experimental setup, in which mildly cystic PKD animals are treated for a short period of time and compared to untreated PKD animals with a similar phenotype, or wildtype animals. This enabled us to identify specific metabolites and genes affected by salsalate. Due to the short period of treatment time, in combination with the mild disease phenotype in which we examined salsalate effects, only mild changes in cellular signaling could reasonably be expected.

At the metabolome level, we found three metabolites significantly changed upon short salsalate treatment via NMR metabolomics: acetylcarnitine, phosphocholine, and tyrosine. Of these three, only acetylcarnitine and phosphocholine show a correction back toward wild-type levels. Acetylcarnitine is the most known derivative of carnitine, an important regulator of fatty acid import into mitochondria. Since the mitochondrial membrane is impermeable to fatty acids (or acyls), acyl-CoA molecules are converted to acyl-carnitines by carnitine palmitoyltransferase to enter the mitochondria for FAO. In addition, acetylcarnitine can be used as an acetyl donor to produce acetyl-CoA. Both processes point toward increased FAO, a process dysregulated in ADPKD.^12^^,^^14^ Also, urinary acetylcarnitine was found to be the best predictor of *Pkd1* mutant status in a juvenile PKD mouse model.^40^

Through the phosphorylation of AMPK,^33^ salsalate induces ACC phosphorylation, thereby inactivating it. When active, ACC produces, via acetyl-CoA carboxylation, the fatty acid import complex inhibitor malonyl-CoA. As salsalate inactivates ACC, this lowers malonyl-CoA levels, thereby lifting FAO inhibition.This regulation has been described extensively in skeletal muscle and adipose tissue research, which already has shown that salsalate can activate FAO *in vitro* and *in vivo.*^41^^,^^42^^,^^43^^,^^44^^,^^45^ In line with these observations, we found multiple genes related to fatty acid metabolism affected by salsalate treatment, including *Aldh1a7, Acss1, Ces2h, Cyp4a14, Ces1e, Vnn1, Hnf4aos*, and *Acot1*, but also genes encoding (fatty acid) transporters, such as *Slc23a3*, *Slc22a29*, and *Abcb1b* (Figures 6B and 6C). Of note here are *Vnn1* and *Hnf4aos*. *Vnn1* has a deviating expression pattern compared to other RNA sequencing hit genes, being upregulated in PKD animals compared to wildtypes, and then also upregulated by short salsalate treatment. *Vnn1* encodes the enzyme pantetheinase, responsible for vitamin B5 (pantothenic acid) recycling.^46^ Vitamin B5 is required for CoA synthesis, which in turn is vital for FAO. As we observe FAO is induced by short salsalate treatment, which requires higher CoA levels; this could explain the curious *Vnn1* expression pattern. *Hnf4aos* encodes a long non-coding RNA with an opposite sequence to the coding sequence for *Hnf4a*, which encodes the transcription factor HNF4α. This transcription factor, downregulated in ADPKD,^40^^,^^47^ has multiple roles in regulating fatty acid metabolism, together with PGC1α, another known transcription factor dysregulated in ADPKD and affected by salsalate treatment.^25^^,^^48^^,^^49^^,^^50^^,^^51^ We observe lower *Hnf4aos* expression with salsalate treatment, and as *Hnf4aos* expression is inversely correlated to *Hnf4a* expression,^52^ this is another way through which salsalate affects fatty acid metabolism. In addition, we also observe an increase in total PGC1α protein levels (Figures 5C and 5D). PGC1α aids in the regulation of fatty metabolism by promoting mitochondrial biogenesis,^53^^,^^54^ which increases mitochondrial content and provides the cell with a higher oxidative capacity. Taken together, we observe on the RNA, protein, and metabolome level that salsalate, through AMPK phosphorylation, can (re-)activate FAO in ADPKD, partly reverting the Warburg-like metabolic reprogramming observed in ADPKD.

Other than acetylcarnitine, we find two other metabolites significantly changed upon short salsalate treatment via NMR metabolomics: phosphocholine and tyrosine (Figures 3B and 3C). Phosphocholine is an intermediate in the Kennedy pathway from choline to phosphatidylcholine, the most abundant phospholipid in mammalian cells. As such, it has a major role in membrane structure and functioning. Phosphatidylcholine is a source for the production of several second messengers, including diacylglycerol, arachidonic acid, and lysophosphatidic acid, thereby affecting multiple signaling pathways that are important mediators of cyst growth, including phosphoinositide/protein kinase C signaling, cyclo-oxygenase, lipo-oxygenase, cytochrome P450 pathways, and G-protein coupled receptor signaling. In recent years, multiple studies have identified increased choline levels, together with increased levels of molecules in the Kennedy pathway, including phosphocholine, as pro-inflammatory factors via macrophage activation.^55^^,^^56^^,^^57^^,^^58^ In ADPKD, increased inflammatory activity, macrophage activation, and influx into the kidney are well-described processes contributing to disease progression, both in preclinical models and patients.^10^^,^^59^ AMPK has also been described to be directly involved in the mediation of inflammatory responses. The well-known pro-inflammatory stimulus tumor necrosis factor alpha (TNF-α) can suppress AMPK activity *in vitro* and *in vivo,*^60^ and the (re-)activation of AMPK has been shown to suppress various inflammatory processes in adipose tissue^61^ and skeletal muscle,^62^ both *in vitro* and *in vivo*. Underlying this regulation is likely the modulation of cellular metabolism by AMPK, which in turn has been shown to affect macrophage activation and polarization.^61^^,^^62^^,^^63^^,^^64^ Thus, as an AMPK activator, salsalate can inhibit inflammatory processes, but salicylate (the active compound of salsalate) has also been described as a direct IKKβ inhibitor, an effector in the pro-inflammatory nuclear factor κB (NF-κB) pathway.^65^^,^^66^^,^^67^ Future studies will be required to delineate the specific targets and pathways that salsalate uses for its anti-inflammatory effects in ADPKD. Although the number of F4/80-positive macrophages is not significantly affected upon short treatment (although a clear non-significant trend is present, Figure S3), salsalate does show anti-inflammatory effects in our study, evidenced by our RNA sequencing data, which show pro-inflammatory genes (*Cxcl10*, *Fcamr Hrg*, *Il1rn*, *Nfil3*) and pathways downregulated upon salsalate treatment (Figures 6B and 6C; Table S9).

Next to changes in energy metabolism and inflammation, RNA sequencing revealed that genes involved in cell proliferation (*Dusp8*, *Eef1akmt3*, *Mcm2*, *Top1mt*, *Top2a*), cell cycle regulation (*Cdca3*, *Cdc20*, *Cdk1*, *Cdkn3*, *Chek1*, *E2f1*, *Pmaip1*, *Wee1*), and mitotic spindle formation (*Cdca8*, *Cenpe*, *Knstrn*, *Ncapg2*, *Nusap1*, *Racgap1*) are affected by short salsalate treatment (Figures 6B and 6C). This was confirmed by immunohistochemical tissue staining for the proliferation marker Ki67 (Figures 7A and 7B). Targeting aberrant proliferation can be beneficial in ADPKD, and we show that salsalate affects cell proliferation *in vivo* relatively soon after administration, suggesting a direct effect. Both salsalate and its active compound salicylate have been identified to affect proliferation in different cell lines.^45^^,^^68^^,^^69^^,^^70^ We also found salsalate to affect cell proliferation in ADPKD in our long treatment study.^25^ Increased cell proliferation has been described extensively as a major driver of cystic disease progression, as the consequence of elevated cAMP levels.^36^^,^^37^^,^^38^ However, we observe that a short salsalate treatment of PKD animals does not affect elevated cAMP levels, indicating the effects elicited by salsalate are cAMP independent (Figure 7D). This must mean that the anti-proliferative effects of salsalate are mediated through another pathway or target. Indeed, AMPK has been shown to inhibit cell proliferation in different models, via the inhibition of *de novo* lipogenesis,^71^^,^^72^^,^^73^^,^^74^ but also through the (in)direct modulation of pro- and anti-proliferative pathways, such as mTOR, Hedgehog, and Hippo, all pathways that have been shown to be overactive in ADPKD.^10^^,^^75^^,^^76^^,^^77^^,^^78^ Of note is that we do not observe increased mTOR signaling in our data, and no salsalate-mediated mTOR inhibition. This is most likely due to the mild disease phenotype which we are studying, in which mTOR signaling is not yet elevated, and on which salsalate does not show any effects yet. This is to be expected, because, due to the mild phenotype, only early and mild changes in cellular signaling will be detected. We have shown before that in a mild PKD phenotype, mTOR signaling is still at wild-type levels.^79^ Other than AMPK, salsalate could affect cell proliferation also via cyclin-dependent kinase 1 or 2 (CDK1/2) interactions. Recently, salicylate (the active compound of salsalate) has been identified to be a binding partner of both CDK1 and CDK2,^80^^,^^81^ suggesting a direct link between salsalate, CDK1/2, and cell proliferation. Interestingly, our RNA sequencing reveals *Cdk1* as one of the genes affected by salsalate, as well as genes encoding several suggested target genes (*Ccnb2*, *Cdca8*, *Cdkn3*, *Wee1*; Figure 6B). *Cdk1* encodes CDK1, a protein that acts as an essential master regulator of cell cycle progression. It was recently reported that CDK1 is an important player in the increased cell proliferation observed in PKD, and that *Cdk1* inactivation improved the cystic phenotype and reduced cell proliferation.^82^ Our RNA sequencing results also reveal other processes that are important in driving disease progression in PKD to be affected by short salsalate treatment, such as oxidative stress caused by mitochondrial dysfunction, associated with lower expression of antioxidant and detoxification enzymes.^18^^,^^19^^,^^83^ We observe several genes encoding detoxification enzymes to be upregulated upon salsalate treatment, such as *Abcb1b*, *Cyp4a14*, *Gstm7*, *Slc17a4*, *Slc23a3*, and *Slc22a9*. This further confirms the versatility that salsalate has in affecting different molecular mechanisms, and its potential as a PKD drug.

To gain more understanding about the metabolic pathways affected in our study, we generated a visual representation of the metabolomic and transcriptomic changes with use of the MRN pathway browser tool.This visualization provides a representation of our findings on a biochemical network and demonstrates that the short salsalate treatment already has multiple effects on the dysregulated PKD metabolome, which is also affecting the genes involved in the metabolic interconversions. The integration of metabolomic and transcriptomic data confirms our findings on the individual metabolite level, as both FAO-related (acetylcarnitine) processes as well as processes related to choline metabolism (phosphocholine) are affected by salsalate. In addition, we observe a mild, but detectable, change in purine metabolism and purine interconversions in the visualization. This is in line with our metabolite-metabolite correlation analyses, in which we found multiple metabolite pairs suggesting that salsalate has an effect on purine metabolism. In particular, metabolite pairs containing IMP came out of our analysis. Purines form the basis of nucleotides, required for DNA synthesis and as such cell proliferation.^84^ In addition to that, purines also have an important role in providing cells with energy (ATP, GTP) and are incorporated within co-factors such as NAD and CoA.^85^ Consequently, there is a close relationship to energy metabolism, with many metabolites used for purine synthesis, and many purine-containing products used in energy metabolism pathways. Because of this, and the metabolic reprogramming observed in ADPKD, it is not surprising that purine metabolism is dysregulated in both juvenile and adult PKD mouse models.^35^^,^^40^^,^^86^ While no causal link has been reported between ADPKD pathogenesis and IMP concentrations, it has recently been shown that CDK7, part of a super-enhancer machinery upregulated in ADPKD, increases kidney IMP concentrations by upregulation of AMP deaminase 3 (AMPD3), and that treatment with the CDK7 inhibitor THZ1 or the AMPD3 inhibitor pentostatin suppresses cyst formation in PKD mouse models.^87^ AMPD3 produces IMP via AMP deamination, lowering AMP levels and, therefore, AMPK activity. As an AMPK activator, salsalate could perhaps re-balance the concentrations of AMP and IMP, explaining the altered metabolite correlation pairs we observe. However, caution is required in interpreting these results, as there is no clear causative link between salsalate treatment and purine metabolism in our data. In addition, the correlation analysis is heavily susceptible to outlier samples that might be driving a significant correlation.

In conclusion, we found that a short treatment of the AMPK activator salsalate in adult, mildly cystic iKspCre-*Pkd1*^del^ mice affects multiple pathways and processes known to be dysregulated in ADPKD, which are major effectors of the disease progression: energy metabolism, cell proliferation, and inflammation. Our combined approach of NMR metabolomics and RNA sequencing analyses show an attenuation of metabolic reprogramming in PKD (Figures 8A and 8B), most notable in acetylcarnitine and phosphocholine concentrations, reflecting increased FAO and reduced pro-inflammatory activity, respectively. Furthermore, RNA sequencing results show salsalate also inhibits cell proliferation, another prominent driver of PKD progression. This was confirmed by immunohistochemical analysis. Metabolite-metabolite correlation analysis showed that salsalate associates with changes in purine metabolism as well. These data give us more insight into the working mechanisms of salsalate in ADPKD, providing a platform for future (pre)clinical investigations.

### Limitations of the study

While we see increased AMPK and ACC phosphorylation in PKD + Salsalate animals, we also observe a similar increase in total AMPK and total ACC protein levels, resulting in unchanged pAMPK/AMPK and pACC/ACC ratios (Figures 5A–5D). Therefore, we cannot provide clear evidence that salsalate induces AMPK & ACC phosphorylation in our study. This can possibly be attributed to the sensitivity of AMPK expression and phosphorylation. As AMPK is an important metabolic sensor, its phosphorylation status, as well as that of direct downstream targets, is very sensitive in stress situations, such as animal sacrifice. However, we do see increased total PGC1α protein expression, which is further downstream of phosphorylated AMPK compared to ACC. However, this provides a small, but limited, confirmation that salsalate does activate AMPK in our model.

Second, we observe a disconnect between RNA sequencing data and protein levels of CDK1 and HMGN2 (Figure S1). The reason behind this is most likely 2-fold. First, as we only are expecting mild changes in cellular signaling, due to the mild disease phenotype and short treatment period, this could result in molecular changes that can be detected at the transcriptomic level but are not yet measurable at the proteomic level. Second, high AMPK activity and low mTOR activity contribute to reduced protein synthesis and inhibition of mRNA translation, via phosphorylation of the eukaryote elongation factor 2 (eEF2) kinase.^88^ As we indeed observe low mTOR activity (Figures 5E and 5F), this could be a viable reason for the disconnect between our transcriptomic and protein data.

Furthermore, anticipating a comment on a limited coverage of the metabolome, we point out that the completeness of the metabolome cannot be covered with a single analytical technique. Every analytical technique gives only a partial overview of the metabolic changes. To this end, in this study, we chose NMR as analytical technique instead of mass spectrometry (MS) because of the superior quantitative capabilities and excellent robustness of NMR. The metabolites (43 and 44 metabolites) in our study might appear to be somehow limited; every single annotated metabolite was not only confidentially identified but also accurately quantified, in contrast to most MS-based metabolomics studies.

## STAR★Methods

### Key resources table

**Table:** 

REAGENT or RESOURCE	SOURCE	IDENTIFIER
**Antibodies**		

Rabbit anti-pAMPK primary antibody	Cell Signaling	2535; RRID: AB_331250
Rabbit anti-AMPK primary antibody	Cell Signaling	2532; RRID: AB_330331
Rabbit anti-pACC primary antibody	Cell Signaling	3661; RRID: AB_330337
Rabbit anti-ACC primary antibody	Cell Signaling	3662; RRID: AB_2219400
Rabbit anti-PGC1α primary antibody	Abcam	ab54481; RRID: AB_881987
Rabbit anti-pS6 primary antibody	Cell Signaling	2215; RRID: AB_331682
Mouse anti-S6 primary antibody	Cell Signaling	2317; RRID: AB_2238583
Rabbit anti-p-p70S6K primary antibody	Cell Signaling	9205; RRID: AB_330944
Rabbit anti-CDK1 primary antibody	Novus Biologicals	NBP1-85729
Rabbit anti-HMGN2 primary antibody	Cell Signaling	9437; RRID: AB_10949505
Mouse anti-CHK1 primary antibody	Santa Cruz	sc-8408; RRID: AB_627257
Mouse anti-Tubulin primary antibody	Calbiochem	CP06; RRID: AB_2617116
Rabbit anti-β-actin primary antibody	Cell Signaling	4967; RRID: AB_330288
Goat-anti-rabbit IRDye 800CW secondary antibody	LI-COR Biosciences	926–32211; RRID: AB_621843
Goat-anti-mouse IRDye 680RD secondary antibody	LI-COR Biosciences	925–68070; RRID: AB_2651128
Rabbit anti-Ki67 primary antibody	Nova Castra	NCL-Ki67p; RRID: AB_442102
Rat anti-F4/80 primary antibody	Serotec	N/A
Rabbit anti-CD3 primary antibody	Abcam	ab16669; RRID: AB_443425
Rat anti-CD19 primary antibody	ThermoFisher Scientific	14-0194-82; RRID: AB_2637171
anti-rat IMMPress™	Vectorlabs	MP-7444; RRID: AB_2336530
anti-rabbit EnVision	Dako	K4011

**Biological samples**		

Mouse kidney samples	This paper	N/A

**Chemicals, peptides, and recombinant proteins**		

Tamoxifen	Sigma-Aldrich	T5648
Salsalate	AK-Scientific	F817
Buffered formaldehyde	In-house	N/A
Methanol LC-MS grade	Merck	1.06035.2500
Sodium azide	Sigma-Aldrich	71289
Trimetilsilyl proprionic-d_4_ sodium salt	Cambridge Isotope Laboratories	DLM-7562-1
Tri-Reagent	Sigma-Aldrich	T9424
FastStart Universal SYBR Green Master (Rox)	Sigma-Aldrich	4913914001
Sodium chloride (NaCl)	Sigma-Aldrich	S3014
EDTA	Sigma-Aldrich	EDS
Sodium deoxycholate (NaDOC)	Sigma-Aldrich	D6750
IGEPAL CA-630	Sigma-Aldrich	I3021
Sodium dodecyl sulfate (SDS)	USB	151-21-3
Protease inhibitor cocktail tablets	Roche Diagnostics	05892970001
Sodium fluoride (NaF)	Sigma-Aldrich	S7920
Sodium orthovanadate (Na_3_VO_4_)	Sigma-Aldrich	S6058
Fish Serum Blocking Buffer	ThermoFisher Scientific	37527
Tween 20	Sigma-Aldrich	T7949

**Critical commercial assays**		

NEBNext Ultra II Directional RNA Library Prep Kit for Illumina	New England BioLabs	E7760
Transcriptor First Strand cDNA Synthesis Kit	Roche Life Science	4897030001
Mouse/Rat cAMP Parameter Assay Kit	R&D Systems	KGE012B

**Deposited data**		

Raw RNA-sequencing data files	This paper	GSE220775

**Experimental models: Organisms/strains**		

tam-*KspCad*-CreER^T2^; *Pkd1del*^*2-*^*^1^*^*l/lox2-11*^	In-house, see ref.^88^ for details	N/A

**Oligonucleotides**		

See Table S10 for primer sequences used		

**Software and algorithms**		

R (version 3.6.3 for metabolomics data analysis, version 4.0.4 for differential gene expression analysis and version 4.2.2 for gene enrichment analysis)	R Core Team and the R Foundation for Statistical Computing	https://www.r-project.org/
Chenomx (version 8.4)	Chenomx NMR suite	https://www.chenomx.com/
BIOWDL RNAseq pipeline v4.1.0	https://zenodo.org/record/3975552	N/A
Image Studio Lite	Licor Bio-Sciences	N/A
ImageJ	NIH	N/A
Adobe Photoshop	Adobe Systems	N/A
GraphPad Prism 8	GraphPad Prism	N/A

**Other**		

Stainless beads (0.2–2.0 mm diameter)	Next Advance	SSB14B
3 mm NMR tubes	Bruker	Z168406
Mouse food pellets containing salsalate	Special Diet Services	N/A
MagNA Lyser Green Beads tubes	Roche Life Science	03358941001
Oligo-dT magnetic beads	New England BioLabs	S1419S
4–20% Criterion™ TGX™ Precast Midi Protein Gel	Bio-Rad Laboratories	5671093/5671095
Trans-Blot Turbo Midi 0.2 μm nitrocellulose Transfer Packs	Bio-Rad Laboratories	1704159
Trans-Blot Turbo Midi 0.2 μm PVDF Transfer Packs	Bio-Rad Laboratories	1704157

### Resource availability

#### Lead contact

Further information and requests for resources and reagents should be directed to and will be fulfilled by the lead contact, Dorien J.M. Peters (d.j.m.peters@lumc.nl), upon reasonable request.

#### Materials availability

• This study did not generate new unique reagents.
• The mouse line used in this study (tam-*KspCad*-CreER^T2^; *Pkd1del*^*2−1L/lox2-11*^) was generated in-house, and is available upon reasonable request from the lead contact with the appropriate Materials Transfer Agreement (MTA).

### Experimental model and study participant details

#### Mouse experimental details

Generation of tamoxifen-inducible kidney-specific *Pkd1* deletion (iKspCre-*Pkd1*^del^) mice (tam-*KspCad*-CreER^T2^; *Pkd1del*^*2−1L/lox2-11*^) was done in-house as described before.^89^ Only adult male mice were used for these experiments, as they display a more severe disease progression compared to adult female mice. All animal experiments were approved by the Animal Experiment Ethics Committee of Leiden University Medical Center and the Commission Biotechnology in Animals of the Dutch Ministry of Agriculture, and performed in accordance to Directive 2010/63/EU for animal experiments.

##### Long treatment

The experimental setup of this mouse study was described before.^25^ In short, iKspCre-*Pkd1*^del^ mice were generated (n = 20 per group), and kidney-specific deletion of the *Pkd1* gene was induced with 150 mg/kg tamoxifen via oral gavage (T5648, Sigma-Aldrich) on postnatal day (PN) 18 and 19. On PN40, treatment with salsalate (#F817, AK-Scientific) was started, at a dose of 400 mg/kg/day (2.5 g/kg of food pellets, Special Diet Services). As a control group, mice which received food pellets generated by the same protocol, but without any drug, were used. Weekly blood urea nitrogen (BUN) measurements using the Reflotron Sprint (Roche Diagnostics) were used to monitor kidney function from PN75. Mice with a BUN >20 mmol/L were considered to have end-stage renal disease (ESRD) and sacrificed. When ±50% of the control mice reached ESRD (at approximately 111–115 days of age), all mice from the control and treatment group were sacrificed.

##### Short treatment

iKspCre-*Pkd1*^del^ mice were generated, and kidney-specific deletion of the *Pkd1* gene was induced with 150 mg/kg tamoxifen via oral gavage (T5648, Sigma-Aldrich) on PN18 and 19. All mice remained on a control diet (without any drug added) until P81. From PN67, weekly BUN measurements were performed to monitor kidney function. Mice with a BUN >20 mmol/L were considered to have reached ESRD and sacrificed. On PN81, mice were split in two groups; one group remained on the control diet for the remainder of the experiment, the other group received salsalate treatment at a dose of 400 mg/kg/day (2.5 g/kg of food pellets, Special Diet Services) for 14 days. After 14 days of treatment (PN95), all mice from both the control and treatment group were sacrificed. Kidneys were removed after sacrifice, weighed for calculation of 2KW/BW%, bisected and then either snap-frozen in liquid nitrogen or fixed in 4% buffered formaldehyde. Snap-frozen tissues were stored at −80°C until further use.

### Method details

#### Metabolite extraction from kidneys

Snap-frozen half kidneys were weighed before being placed in pre-weighed microtubes containing stainless beads (0.9–2.0 mm mix). 3 volumes of LC-MS quality dry-ice cold methanol was added (3:1 methanol-to-kidney weight) for sample homogenization and allow for metabolite extraction in the same step. Samples were further homogenized in a Bullet Blender 24 (Next Advance Inc., NY, USA) for 1 min at max. speed and were placed back on dry ice for 1 min to maintain samples at low temperature. This cycle was conducted up to 3 times until the kidneys were fully homogenized. Homogenates were centrifuged (16000 x *g*, 20 min, 4°C) and the supernatant was taken to a new microtube. Methanol extraction was repeated one more time and supernatants were combined. The resulting non-soluble pellet from the extraction was dried in a SpeedVac until fully dried and was subsequently weighed for further normalization. Combined supernatants were also dried in the SpeedVac and were kept at −80°C until the day of analysis. Prior to NMR analysis, dried extractants were reconstituted in 280 μL 150 mM phosphate buffer (pH 7.4) in deuterated water including 0.2 mM NaN_3_ and 0.4 mM trimetilsilyl proprionic-d_4_ sodium salt (TSP, Cambridge Isotope Laboratories, Inc.) as internal standard used for NMR referencing and quantification. A quality control (QC) sample was made by aliquoting the same volume of each sample. 200 μL per sample were placed into a 96 well plate and 180 μL were transferred to 3 mm tubes by a Gilson 215 liquid handler.

#### NMR metabolomics

^1^H-NMR spectra of all samples were obtained on a 14.1 T (600 MHz) Bruker Avance II NMR spectrometer, using a *noesygppr1d* pulse sequence (Topspin v3.0, Bruker Biospin Ltd, Karlsruhe, Germany). Each spectrum was imported to Chenomx NMR suite 8.4 (Chenomx NMR suite, v8.0, Edmonton, AB, Canada) for the quantification, in mmol/L, of the different metabolites by integration of its proton resonances. For each metabolite, concentration was converted from mmol/L to μg/mg of insoluble pellet by using the weight of the non-soluble pellet, the molecular weight and the extractant solvent. The data was further normalized using probabilistic quotient normalization (PQN).^90^ All the p values from the Mann-Whitney U-test shown are non-corrected for multiple testing because the changes induced by salsalate are only mild in nature, as evidenced by the small log_2_ fold changes of the significantly changed metabolites. In addition, since our study is inherently exploratory, aiming to identify potential patterns and generate hypotheses rather than to confirm pre-established assumptions or theories, failing to detect a true effect (Type II error) was a greater concern than detecting a false positive (Type I error).

#### RNA isolation and sequencing

Kidney homogenates were prepared from snap-frozen tissues with a MagNA Lyser instrument (Roche Life Science) in MagNA Lyser Green Beads tubes (Roche Life Science). Total RNA was then isolated from the homogenates using Tri-Reagent (Sigma-Aldrich). RNA-sequencing was performed on the Illumina NovaSeq6000, performed at GenomeScan (Leiden, The Netherlands). The NEBNext Ultra II Directional RNA Library Prep Kit for Illumina was used for sample processing. Briefly, mRNA was isolated from total RNA using the oligo-dT magnetic beads. After fragmentation of the mRNA, a cDNA synthesis was performed. This was used for ligation with the sequencing adapters and PCR amplification of the resulting product. The quality and yield after sample preparation was measured with the Fragment Analyzer. The size of the resulting products was consistent with the expected size distribution (a broad peak between 300 and 500 bp). Clustering and DNA sequencing using the NovaSeq6000 was performed according to manufacturer’s protocols. A concentration of 1.1 nM of DNA was used. Image analysis, base calling, and quality check was performed with the Illumina data analysis pipeline RTA3.4.4 and Bcl2fastq v2.20. All samples had a quality score Q30 for more than 90.79% of reads. Sequencing files were then processed using the opensource BIOWDL RNAseq pipeline v4.1.0 (https://zenodo.org/record/3975552) developed at the LUMC. This pipeline performs FASTQ pre-processing (including quality control, quality trimming, and adapter clipping), RNAseq read alignment, read quantification, and optionally transcript assembly. FastQC was used for checking raw read QC. Adapter clipping was performed using Cutadapt (v2.10) with default settings. RNAseq read alignment was performed using STAR (v2.7.5a) on GRCm39 mouse reference genome (Ensembl v103). The gene read quantification was performed using HTSeq-count (v0.12.4) with setting “–stranded = yes”. The gene annotation used for quantification was Ensembl version 103. Using the gene read count matrix, CPM was calculated per sample on all annotated genes.

#### Differential gene expression analysis

R v4.0.4 is used for the differential gene expression analysis. The read count data of 32 samples are labeled into three groups: Wildtype, PKD & PKD + Salsalate. Low expressed genes have been removed during pre-processing. Only genes with a Log2CPM cut-off of 1 in at least 25% of the samples are kept. Next, TMM normalization is performed on the genes that remain. All three groups are compared against each other using EdgeR v3.32.1, in the following comparisons: (1) PKD vs. Wildtype, (2) PKD + Salsalate vs. Wildtype & (3) PKD + Salsalate vs. PKD. All genes with a p value (FDR) < 0.05 are declared significant.

#### Gene enrichment analysis

Using the output of the gene expression comparison "PKD + Salsalate vs. PKD", an enrichment analysis (ORA) is performed using R v4.2.2. The genes are selected for both Log2FC (absolute Log2FC > 0.5) and/or FDR <0.05. This resulted in 184 genes (55 upregulated & 129 downregulated). Next, these genes were used as input to gProfiler2 v0.2.1, using three different databases: (1) GO (MF, CC & BP), (2) KEGG & (3) Reactome. All terms with a p value (FDR) < 0.05 are declared significant.

#### Quantitative PCR

Total RNA was reverse transcribed to cDNA with the Transcriptor First Strand cDNA Synthesis Kit (Roche Life Science), and qPCR was performed using the FastStart Universal SYBR Green Master (Rox) (Sigma-Aldrich), according to the manufacturer’s protocol. mRNA expression was normalized to *Hprt* expression and expressed as a fold change using the ΔΔCT method. The primer sequences used are listed in the key resources table.

#### Protein isolation and Western blotting

Snap-frozen tissues were homogenized in RIPA buffer (50 mM Tris-HCl pH 7.4, 150 mM NaCl, 1 mM EDTA, 0.5% NaDOC, 1% IGEPAL CA-630, 0.1% SDS), supplemented with protease and phosphatase inhibitors (50 mM NaF, 1 mM Na_3_VO_4_, and protease inhibitor cocktail (#05892970001, Roche Diagnostics)) using MagNA Lyser technology. Lysates were stored at −80°C until further use. Proteins were separated via SDS-PAGE using 4–20% Criterion TGX Precast Midi Protein Gels (Bio-Rad Laboratories, Veenendaal, The Netherlands), followed by transfer to a nitrocellulose or PVDF membrane (Bio-Rad Laboratories). Membranes were blocked for 1 h at room temperature in 25% Fish Serum Blocking Buffer (37527, ThermoFisher Scientific, Rockford, IL, United States) in Tris-buffered saline (TBS), followed by overnight incubation at 4°C with primary antibodies against pAMPK (2535, Cell Signaling), AMPK (2532, Cell Signaling), pACC (3661, Cell Signaling), ACC (3662, Cell Signaling), PGC1α (ab54481, Abcam), pS6 (2215, Cell Signaling), S6 (2317, Cell Signaling), p-p70S6K (9205, Cell Signaling), CDK1 (NBP1-85729, Novus Biologicals), HMGN2 (9437, Cell Signaling), tubulin (CP06, Calbiochem) or β-actin (4967, Cell Signaling). Blots were then washed with 0.1% Tween 20 (T7949, Sigma-Aldrich) in TBS and incubated for 1 h at room temperature with goat-anti-rabbit IRDye 800CW (926–32211, LI-COR Biosciences) or goat-anti-mouse IRDye 680RD (925–68070, LI-COR Biosciences) secondary antibody. Blots were visualized and scanned with the Odyssey CLx Imaging System (Li-COR Biosciences). Protein content was quantified via densitometric analysis (Image Studio Lite, Li-COR Biosciences), normalized to tubulin or β-actin protein content and expressed as a fold change.

#### Histology and immunohistochemistry

Tissues fixed overnight in 4% buffered formaldehyde were embedded in paraffin. Ki67 (as a cell proliferation marker), CD3 (as a T cell marker), CD19 (as a B cell marker) and F4/80 (to detect macrophages) stainings were performed, as described before.^25^^,^^79^ Briefly, antigen retrieval was done with 10 mM citrate buffer (Ki67, CD3, CD19) or proteinase K (F4/80, Dako), after which endogenous peroxidase blocking was done via 20 min incubation with 0.12% H_2_O_2_. Then, tissue sections were incubated with rabbit anti-Ki67 (1:2000, Nova Castra) or rat anti-F4/80 (1:100, Serotec), followed by a secondary incubation with anti-rat IMMPress (Vectorlabs) or anti-rabbit EnVision (Dako). Immune reactions were then revealed using diaminobenzidine as a chromogen and counterstained with hematoxylin. Sections were then dehydrated and mounted. Quantification was done using Photoshop software (Adobe) as described before.^91^ A color pallet specific to the brown F4/80 signal was designed. Then, the total number of pixels from the F4/80 signal and the total number of pixels from the entire section (excluding cysts) was used to calculate the F4/80^+^ percentage within the section.

#### Quantification of Ki67 staining

The percentage of Ki67^+^-nuclei was calculated using ImageJ software (NIH). 10 random, non-overlapping sections of the kidney were counted per mouse. First, the image was split into its blue component (all nuclei) and its brown component (Ki67^+^ nuclei), after which a minimal threshold value was set, that was used for all pictures. Then, the number of Ki67^+^-nuclei and the number of total nuclei was used to calculate the Ki67^+^ percentage within the section.

#### Cyst diameter quantification

The cyst/tubule diameter was calculated using ImageJ software. 10 random, non-overlapping sections were counted per mouse. Each cyst or tubule within the section was manually judged, and subsequently, the number of Ki67^+^-nuclei per cyst or tubule was counted. The cystic/tubular diameter was measured in pixels and data was divided in four groups: tubules, small cysts (<1.75x average tubular diameter), medium cysts (1.75-3x average tubular diameter) and large cysts (>3x tubular diameter).

#### cAMP assay

Kidney tissue cAMP levels were measured using the Mouse/Rat cAMP Parameter Assay Kit In short, snap-frozen tissues were homogenized in 0.1 N HCl (1:5 w/v ratio) and then centrifuged at 10000 *x g* for 10 min. The supernatant was then taken and neutralized with 1 N NaOH. The assay protocol was then performed, according to the manufacturer instructions.

#### Network analysis

Network analysis was performed to visualize the measured metabolites and their biochemical relationships in a holistic fashion. For this purpose we developed the Mouse Reaction Network (MRN), a curated version of the Mouse1 genome-scale metabolic model (GSMM)^92^ that we enriched with compound synonyms and external identifiers from the Chemical Entities of Biological Interest (ChEBI)^93^ and Ensembl gene and transcript identifiers from GENCODE release M30.^94^ Metabolites identified in the -omics analyses were mapped on the curated model and subsequently biochemical paths between the metabolites were determined consisting of one or two reaction steps, using a generic path finding algorithm that was developed in-house. Since our NMR platform covered mostly metabolites from central metabolism, we also added metabolites from glycolysis and the Krebs cycle that could not be quantified in order to prevent gaps in the traditional pathways. To ensure that the reaction paths represented relevant biochemical conversions, each path was checked for stoichiometric and thermodynamic consistency. In addition, only substrate-product mappings were considered that involved the transfer of carbon-based moieties, except for SAM which was considered a hub metabolite. As a consequence, half-reactions involving the transfer of electrons, amino or phosphate groups were decoupled from the main reaction in the path finding procedure. For example, in the reaction NADH + pyruvate <=> NAD+ + lactate, only NADH and NAD+ are linked in the network and pyruvate and lactate are linked. Likewise, in the reaction glutamate + pyruvate <=> AKG + alanine, only glutamate and AKG are linked, and pyruvate and alanine are linked. In this way, we prevented the creation of a crowded, highly connected network in which all species are interconnected via a few hub metabolites such as H^+^, H_2_O, ATP and NADH. Finally, transporter reactions that transferred compounds over the cellular membranes and (half)reactions that involved uniquely produced metabolites were given a weight of zero in the path finding procedure.

In addition to the network analysis of the NMR metabolites, we repeated the analysis by including metabolites that were likely affected by changes in enzyme activity due to the altered transcript levels. In order to determine a metabolite list from the transcriptomics data we used a modified version of the Reporter Metabolites method.^95^ Specifically, for each reaction we determined all substrate-product pairs through which a path could occur, and mapped the gene identifiers of the enzymes catalyzing each reaction to the corresponding metabolite pairs. This resulted in a mapping between genes and the metabolite ratios they could potentially affect. Since some ratios occurred in many reactions, such as ATP/ADP, while others were specific to a single reaction, we calculated weights to quantify the expected amount of influence each gene had on the linked metabolite ratios. Specifically, we first set each weight to one, then normalized weights on the number of genes connected to each ratio, and then normalized the weights on the maximum weight per gene. For each metabolite ratio, the weighted Fisher’s method was then used to get a measure of the extent to which sum of the altered transcript levels would affect the ratio:

$$X_{i}=-\sum_{j}w_{\text{ij}}\;\log\left(p_{j}\right)$$

X_i_ = -Σ_j_ w_ij_ log(p_j_)where X_i_ is the weighted Fisher’s statistic for ratio i, w_ij_ is the weight of gene j for ratio i, and p_j_ is the rank-based inverse uniform transformed p value of transcript j. The weighted Fisher’s statistic X_i_ was then tested against a null distribution that was simulated for each ratio (N = 100000) in order to estimate the ratio’s p value. All metabolites in ratios with an estimated p < 0.05 were then included in the path analysis. Since the resulting network contained many small subnetworks, often consisting of only a single metabolic reaction, for the final visualization we filtered out all subnetworks that consisted of only one or two significant genes and/or metabolites. The resulting metabolic networks were integrated with the MRN knowledge base and the omics data and were subsequently exported as an interactive HTML/JavaScript document to facilitate the inspection of the results. To improve readability, we filtered the omics data table in the HTML/JavaScript document for genes and metabolites that were significantly altered after correction for multiple testing. All computations were performed in MATLAB 2019b.

### Quantification and statistical analysis

Statistical & data analyses were performed with GraphPad Prism 8 (GraphPad Software) or R. All results are expressed as mean ± SD, unless stated otherwise in the figure legends. Comparisons between two groups were done using the two-tailed unpaired Student’s *t* test (normal distributed data) or the Mann-Whitney U-test (non-normal distributed data), while comparisons between multiple groups were done using the one-way ANOVA, followed by Tukey’s or Dunnett’s multiple comparison test. Metabolite-metabolite correlations were done via Pearson’s correlation; first, per experimental group (wildtype, PKD and PKD+SAL). Then, only those correlations that were statistically significant (p < 0.05) in each of the two groups involved in the different comparisons (PKD vs. PKD+SAL and PKD vs. wildtype) were kept. Results were considered statistically significant when p < 0.05 or FDR <0.05. ∗p < 0.05, ∗∗p < 0.01, ∗∗∗p < 0.001, ∗∗∗∗p < 0.0001.

## Data Availability

• Data: RNA-sequencing data have been deposited at the Gene Expression Omnibus (GEO) and are publicly available under accession number GSE220775. The Mouse Reaction Network (MRN) integrated data visualisation is available at https://lumc.github.io/MRN-browser/salsalate-multiomics/. Other data reported in this paper will be shared by the lead contact upon reasonable request. • Code: This paper does not report any original code. • Any additional information required to reanalyze the data reported in this paper is available from the lead contact upon reasonable request.

## Supplemental information

Document S1. Figures S1–S7 and Tables S3 and S10

Table S1. Kidney metabolite concentrations of long treatment salsalate study with descriptive statistics related to Figure 1 & Table 1 After Pkd1 deletion on PN18, mice received salsalate treatment from PN40 until 50% of the control group (no treatment) reached ESRD. Kidneys were then bisected and snap-frozen in liquid nitrogen. Metabolites were extracted in methanol (3:1 ratio to KW), after which 1H-NMR metabolomics was performed. 43 metabolites were detectable and quantifiable. Data are shown as mean ± SD. ∗p < 0.05, ∗∗p < 0.01, ∗∗∗p < 0.001 vs. wild type. #p < 0.05, ##p < 0.01, ###p < 0.001 vs. PKD, measured by Mann-Whitney U non-parametric univariate test.

Table S2. Comparison of published PKD metabolomic datasets related to Figure 1

Table S4. Kidney metabolite concentrations of short treatment salsalate study with descriptive statistics related to Figure 2 & Table 2 After Pkd1 deletion on PN18, mice remained untreated until PN81, at which point half of the mice received salsalate treatment for 14 days, and the other half remained untreated. Kidneys were then bisected and snap-frozen in liquid nitrogen. Metabolites were extracted in methanol (3:1 ratio to KW), after which 1H-NMR metabolomics was performed. 44 metabolites were detectable and quantifiable. Data are shown as mean ± SD. ∗p < 0.05, ∗∗p < 0.01, ∗∗∗p < 0.001 vs. wildtype. #p < 0.05, ##p < 0.01, ###p < 0.001 vs. PKD, measured by Mann-Whitney U non-parametric univariate test.

Table S5. Pearson’s correlation values for all metabolite pairs in the wildtype group related to Figures 3 and 4

Table S6. Pearson’s correlation values for all metabolite pairs in the PKD group related to Figures 3 and 4

Table S7. Pearson’s correlation values for all metabolite pairs in the PKD + Salsalate group related to Figures 3 and 4

Table S8. Gene set enrichment analysis (GSEA) results for upregulated genes found between PKD + Salsalate & PKD related to Figure 6 Genes selected for GSEA either had FDR <0.05 and/or Log2FC < 0.5 / > 0.5. This selection resulted in 184 genes, of which 55 were upregulated by short salsalate treatment. These genes were used as input to gProfiler2 v0.2.1, using three different datasets: (1) GO, (2) KEGG and (3) Reactome. All terms with a p value (FDR) p < 0.05 are declared significant.

Table S9. Gene set enrichment analysis (GSEA) results for downregulated genes found between PKD + Salsalate & PKD related to Figure 6 Genes selected for GSEA either had FDR <0.05 and/or Log2FC < 0.5 / > 0.5. This selection resulted in 184 genes, of which 129 were downregulated by short salsalate treatment. These genes were used as input to gProfiler2 v0.2.1, using three different datasets: (1) GO, (2) KEGG and (3) Reactome. All terms with a p value (FDR) p < 0.05 are declared significant.

## References

[1] Bergmann C., Guay-Woodford L.M., Harris P.C., Horie S., Peters D.J.M., Torres V.E. (2018). Polycystic kidney disease. Nat. Rev. Dis. Prim..

[2] Stillman B. (1994). The polycystic kidney disease 1 gene encodes a 14 kb transcript and lies within a duplicated region on chromosome 16. The European Polycystic Kidney Disease Consortium. Cell.

[3] Mochizuki T., Wu G., Hayashi T., Xenophontos S.L., Veldhuisen B., Saris J.J., Reynolds D.M., Cai Y., Gabow P.A., Pierides A. (1996). PKD2, a gene for polycystic kidney disease that encodes an integral membrane protein. Science.

[4] Yu Y., Ulbrich M.H., Li M.H., Buraei Z., Chen X.Z., Ong A.C.M., Tong L., Isacoff E.Y., Yang J. (2009). Structural and molecular basis of the assembly of the TRPP2/PKD1 complex. Proc. Natl. Acad. Sci. USA.

[5] Su Q., Hu F., Ge X., Lei J., Yu S., Wang T., Zhou Q., Mei C., Shi Y. (2018). Structure of the human PKD1-PKD2 complex. Science.

[6] Wang Z., Ng C., Liu X., Wang Y., Li B., Kashyap P., Chaudhry H.A., Castro A., Kalontar E.M., Ilyayev L. (2019). The ion channel function of polycystin-1 in the polycystin-1/polycystin-2 complex. EMBO Rep..

[7] Ha K., Nobuhara M., Wang Q., Walker R.V., Qian F., Schartner C., Cao E., Delling M. (2020). The heteromeric PC-1/PC-2 polycystin complex is activated by the PC-1 N-terminus. Elife.

[8] Shen P.S., Yang X., DeCaen P.G., Liu X., Bulkley D., Clapham D.E., Cao E. (2016). The Structure of the Polycystic Kidney Disease Channel PKD2 in Lipid Nanodiscs. Cell.

[9] Grieben M., Pike A.C.W., Shintre C.A., Venturi E., El-Ajouz S., Tessitore A., Shrestha L., Mukhopadhyay S., Mahajan P., Chalk R. (2017). Structure of the polycystic kidney disease TRP channel Polycystin-2 (PC2). Nat. Struct. Mol. Biol..

[10] Formica C., Peters D.J.M. (2020). Molecular pathways involved in injury-repair and ADPKD progression. Cell. Signal..

[11] Rowe I., Chiaravalli M., Mannella V., Ulisse V., Quilici G., Pema M., Song X.W., Xu H., Mari S., Qian F. (2013). Defective glucose metabolism in polycystic kidney disease identifies a new therapeutic strategy. Nat. Med..

[12] Menezes L.F., Lin C.C., Zhou F., Germino G.G. (2016). Fatty Acid Oxidation is Impaired in An Orthologous Mouse Model of Autosomal Dominant Polycystic Kidney Disease. EBioMedicine.

[13] Padovano V., Kuo I.Y., Stavola L.K., Aerni H.R., Flaherty B.J., Chapin H.C., Ma M., Somlo S., Boletta A., Ehrlich B.E. (2017). The polycystins are modulated by cellular oxygen-sensing pathways and regulate mitochondrial function. Mol. Biol. Cell.

[14] Podrini C., Rowe I., Pagliarini R., Costa A.S.H., Chiaravalli M., Di Meo I., Kim H., Distefano G., Tiranti V., Qian F. (2018). Dissection of metabolic reprogramming in polycystic kidney disease reveals coordinated rewiring of bioenergetic pathways. Commun. Biol..

[15] Flowers E.M., Sudderth J., Zacharias L., Mernaugh G., Zent R., DeBerardinis R.J., Carroll T.J. (2018). Lkb1 deficiency confers glutamine dependency in polycystic kidney disease. Nat. Commun..

[16] Soomro I., Sun Y., Li Z., Diggs L., Hatzivassiliou G., Thomas A.G., Rais R., Parker S.J., Slusher B.S., Kimmelman A.C. (2018). Glutamine metabolism via glutaminase 1 in autosomal-dominant polycystic kidney disease. Nephrol. Dial. Transplant..

[17] Trott J.F., Hwang V.J., Ishimaru T., Chmiel K.J., Zhou J.X., Shim K., Stewart B.J., Mahjoub M.R., Jen K.Y., Barupal D.K. (2018). Arginine reprogramming in ADPKD results in arginine-dependent cystogenesis. Am. J. Physiol. Renal Physiol..

[18] Ishimoto Y., Inagi R., Yoshihara D., Kugita M., Nagao S., Shimizu A., Takeda N., Wake M., Honda K., Zhou J., Nangaku M. (2017). Mitochondrial Abnormality Facilitates Cyst Formation in Autosomal Dominant Polycystic Kidney Disease. Mol. Cell Biol..

[19] Lin C.C., Kurashige M., Liu Y., Terabayashi T., Ishimoto Y., Wang T., Choudhary V., Hobbs R., Liu L.K., Lee P.H. (2018). A cleavage product of Polycystin-1 is a mitochondrial matrix protein that affects mitochondria morphology and function when heterologously expressed. Sci. Rep..

[20] Chiaravalli M., Rowe I., Mannella V., Quilici G., Canu T., Bianchi V., Gurgone A., Antunes S., D'Adamo P., Esposito A. (2016). 2-Deoxy-d-Glucose Ameliorates PKD Progression. J. Am. Soc. Nephrol..

[21] Warner G., Hein K.Z., Nin V., Edwards M., Chini C.C.S., Hopp K., Harris P.C., Torres V.E., Chini E.N. (2016). Food Restriction Ameliorates the Development of Polycystic Kidney Disease. J. Am. Soc. Nephrol..

[22] Kipp K.R., Rezaei M., Lin L., Dewey E.C., Weimbs T. (2016). A mild reduction of food intake slows disease progression in an orthologous mouse model of polycystic kidney disease. Am. J. Physiol. Renal Physiol..

[23] Hajarnis S., Lakhia R., Yheskel M., Williams D., Sorourian M., Liu X., Aboudehen K., Zhang S., Kersjes K., Galasso R. (2017). microRNA-17 family promotes polycystic kidney disease progression through modulation of mitochondrial metabolism. Nat. Commun..

[24] Torres J.A., Kruger S.L., Broderick C., Amarlkhagva T., Agrawal S., Dodam J.R., Mrug M., Lyons L.A., Weimbs T. (2019). Ketosis Ameliorates Renal Cyst Growth in Polycystic Kidney Disease. Cell Metab..

[25] Leonhard W.N., Song X., Kanhai A.A., Iliuta I.A., Bozovic A., Steinberg G.R., Peters D.J.M., Pei Y. (2019). Salsalate, but not metformin or canagliflozin, slows kidney cyst growth in an adult-onset mouse model of polycystic kidney disease. EBioMedicine.

[26] Torres V.E., Chapman A.B., Devuyst O., Gansevoort R.T., Grantham J.J., Higashihara E., Perrone R.D., Krasa H.B., Ouyang J., Czerwiec F.S., TEMPO 3:4 Trial Investigators (2012). Tolvaptan in patients with autosomal dominant polycystic kidney disease. N. Engl. J. Med..

[27] Torres V.E., Chapman A.B., Devuyst O., Gansevoort R.T., Perrone R.D., Koch G., Ouyang J., McQuade R.D., Blais J.D., Czerwiec F.S. (2017). Tolvaptan in Later-Stage Autosomal Dominant Polycystic Kidney Disease. N. Engl. J. Med..

[28] Takiar V., Nishio S., Seo-Mayer P., King J.D., Li H., Zhang L., Karihaloo A., Hallows K.R., Somlo S., Caplan M.J. (2011). Activating AMP-activated protein kinase (AMPK) slows renal cystogenesis. Proc. Natl. Acad. Sci. USA.

[29] Lian X., Wu X., Li Z., Zhang Y., Song K., Cai G., Li Q., Lin S., Chen X., Bai X.Y. (2019). The combination of metformin and 2-deoxyglucose significantly inhibits cyst formation in miniature pigs with polycystic kidney disease. Br. J. Pharmacol..

[30] Pastor-Soler N.M., Li H., Pham J., Rivera D., Ho P.Y., Mancino V., Saitta B., Hallows K.R. (2022). Metformin improves relevant disease parameters in an autosomal dominant polycystic kidney disease mouse model. Am. J. Physiol. Renal Physiol..

[31] Dagorn P.G., Buchholz B., Kraus A., Batchuluun B., Bange H., Blockken L., Steinberg G.R., Moller D.E., Hallakou-Bozec S. (2023). A novel direct adenosine monophosphate kinase activator ameliorates disease progression in preclinical models of Autosomal Dominant Polycystic Kidney Disease. Kidney Int..

[32] Amann R., Peskar B.A. (2002). Anti-inflammatory effects of aspirin and sodium salicylate. Eur. J. Pharmacol..

[33] Hawley S.A., Fullerton M.D., Ross F.A., Schertzer J.D., Chevtzoff C., Walker K.J., Peggie M.W., Zibrova D., Green K.A., Mustard K.J. (2012). The ancient drug salicylate directly activates AMP-activated protein kinase. Science.

[34] Ramalingam H., Kashyap S., Cobo-Stark P., Flaten A., Chang C.M., Hajarnis S., Hein K.Z., Lika J., Warner G.M., Espindola-Netto J.M. (2021). A methionine-Mettl3-N(6)-methyladenosine axis promotes polycystic kidney disease. Cell Metab..

[35] Hopp K., Kleczko E.K., Gitomer B.Y., Chonchol M., Klawitter J., Christians U., Klawitter J. (2022). Metabolic reprogramming in a slowly developing orthologous model of polycystic kidney disease. Am. J. Physiol. Renal Physiol..

[36] Hanaoka K., Guggino W.B. (2000). cAMP regulates cell proliferation and cyst formation in autosomal polycystic kidney disease cells. J. Am. Soc. Nephrol..

[37] Yamaguchi T., Nagao S., Wallace D.P., Belibi F.A., Cowley B.D., Pelling J.C., Grantham J.J. (2003). Cyclic AMP activates B-Raf and ERK in cyst epithelial cells from autosomal-dominant polycystic kidneys. Kidney Int..

[38] Sussman C.R., Wang X., Chebib F.T., Torres V.E. (2020). Modulation of polycystic kidney disease by G-protein coupled receptors and cyclic AMP signaling. Cell. Signal..

[39] Kanhai A.A., Bange H., Verburg L., Dijkstra K.L., Price L.S., Peters D.J.M., Leonhard W.N. (2020). Renal cyst growth is attenuated by a combination treatment of tolvaptan and pioglitazone, while pioglitazone treatment alone is not effective. Sci. Rep..

[40] Menezes L.F., Zhou F., Patterson A.D., Piontek K.B., Krausz K.W., Gonzalez F.J., Germino G.G. (2012). Network analysis of a Pkd1-mouse model of autosomal dominant polycystic kidney disease identifies HNF4α as a disease modifier. PLoS Genet..

[41] Meex R.C.R., Phielix E., Moonen-Kornips E., Schrauwen P., Hesselink M.K.C. (2011). Stimulation of human whole-body energy expenditure by salsalate is fueled by higher lipid oxidation under fasting conditions and by higher oxidative glucose disposal under insulin-stimulated conditions. J. Clin. Endocrinol. Metab..

[42] van Dam A.D., Nahon K.J., Kooijman S., van den Berg S.M., Kanhai A.A., Kikuchi T., Heemskerk M.M., van Harmelen V., Lombès M., van den Hoek A.M. (2015). Salsalate Activates Brown Adipose Tissue in Mice. Diabetes.

[43] Liang W., Verschuren L., Mulder P., van der Hoorn J.W.A., Verheij J., van Dam A.D., Boon M.R., Princen H.M.G., Havekes L.M., Kleemann R., van den Hoek A.M. (2015). Salsalate attenuates diet induced non-alcoholic steatohepatitis in mice by decreasing lipogenic and inflammatory processes. Br. J. Pharmacol..

[44] Nie L., Yuan X.L., Jiang K.T., Jiang Y.H., Yuan J., Luo L., Cui S.W., Sun C. (2017). Salsalate Activates Skeletal Muscle Thermogenesis and Protects Mice from High-Fat Diet Induced Metabolic Dysfunction. EBioMedicine.

[45] Day E.A., Ford R.J., Smith B.K., Houde V.P., Stypa S., Rehal S., Lhotak S., Kemp B.E., Trigatti B.L., Werstuck G.H. (2021). Salsalate reduces atherosclerosis through AMPKβ1 in mice. Mol. Metabol..

[46] Pitari G., Malergue F., Martin F., Philippe J.M., Massucci M.T., Chabret C., Maras B., Duprè S., Naquet P., Galland F. (2000). Pantetheinase activity of membrane-bound Vanin-1: lack of free cysteamine in tissues of Vanin-1 deficient mice. FEBS Lett..

[47] Song X., Di Giovanni V., He N., Wang K., Ingram A., Rosenblum N.D., Pei Y. (2009). Systems biology of autosomal dominant polycystic kidney disease (ADPKD): computational identification of gene expression pathways and integrated regulatory networks. Hum. Mol. Genet..

[48] Hayhurst G.P., Lee Y.H., Lambert G., Ward J.M., Gonzalez F.J. (2001). Hepatocyte nuclear factor 4alpha (nuclear receptor 2A1) is essential for maintenance of hepatic gene expression and lipid homeostasis. Mol. Cell Biol..

[49] Rhee J., Ge H., Yang W., Fan M., Handschin C., Cooper M., Lin J., Li C., Spiegelman B.M. (2006). Partnership of PGC-1alpha and HNF4alpha in the regulation of lipoprotein metabolism. J. Biol. Chem..

[50] Dankel S.N., Hoang T., Flågeng M.H., Sagen J.V., Mellgren G. (2010). cAMP-mediated regulation of HNF-4alpha depends on the level of coactivator PGC-1alpha. Biochim. Biophys. Acta.

[51] Yan Y., Yang X., Zhao T., Zou Y., Li R., Xu Y. (2017). Salicylates promote mitochondrial biogenesis by regulating the expression of PGC-1α in murine 3T3-L1 pre-adipocytes. Biochem. Biophys. Res. Commun..

[52] Lindeboom R.G., van Voorthuijsen L., Oost K.C., Rodríguez-Colman M.J., Luna-Velez M.V., Furlan C., Baraille F., Jansen P.W., Ribeiro A., Burgering B.M. (2018). Integrative multi-omics analysis of intestinal organoid differentiation. Mol. Syst. Biol..

[53] Steinberg G.R., Carling D. (2019). AMP-activated protein kinase: the current landscape for drug development. Nat. Rev. Drug Discov..

[54] Fan W., Evans R. (2015). PPARs and ERRs: molecular mediators of mitochondrial metabolism. Curr. Opin. Cell Biol..

[55] Snider S.A., Margison K.D., Ghorbani P., LeBlond N.D., O'Dwyer C., Nunes J.R.C., Nguyen T., Xu H., Bennett S.A.L., Fullerton M.D. (2018). Choline transport links macrophage phospholipid metabolism and inflammation. J. Biol. Chem..

[56] Que X., Hung M.Y., Yeang C., Gonen A., Prohaska T.A., Sun X., Diehl C., Määttä A., Gaddis D.E., Bowden K. (2018). Oxidized phospholipids are proinflammatory and proatherogenic in hypercholesterolaemic mice. Nature.

[57] Sanchez-Lopez E., Zhong Z., Stubelius A., Sweeney S.R., Booshehri L.M., Antonucci L., Liu-Bryan R., Lodi A., Terkeltaub R., Lacal J.C. (2019). Choline Uptake and Metabolism Modulate Macrophage IL-1β and IL-18 Production. Cell Metab..

[58] Di Gioia M., Spreafico R., Springstead J.R., Mendelson M.M., Joehanes R., Levy D., Zanoni I. (2020). Endogenous oxidized phospholipids reprogram cellular metabolism and boost hyperinflammation. Nat. Immunol..

[59] Song C.J., Zimmerman K.A., Henke S.J., Yoder B.K. (2017). Inflammation and Fibrosis in Polycystic Kidney Disease. Results Probl. Cell Differ..

[60] Steinberg G.R., Michell B.J., van Denderen B.J.W., Watt M.J., Carey A.L., Fam B.C., Andrikopoulos S., Proietto J., Görgün C.Z., Carling D. (2006). Tumor necrosis factor α-induced skeletal muscle insulin resistance involves suppression of AMP-kinase signaling. Cell Metab..

[61] Galic S., Fullerton M.D., Schertzer J.D., Sikkema S., Marcinko K., Walkley C.R., Izon D., Honeyman J., Chen Z.P., van Denderen B.J. (2011). Hematopoietic AMPK β1 reduces mouse adipose tissue macrophage inflammation and insulin resistance in obesity. J. Clin. Invest..

[62] Mounier R., Théret M., Arnold L., Cuvellier S., Bultot L., Göransson O., Sanz N., Ferry A., Sakamoto K., Foretz M. (2013). AMPKα1 Regulates Macrophage Skewing at the Time of Resolution of Inflammation during Skeletal Muscle Regeneration. Cell Metab..

[63] Sag D., Carling D., Stout R.D., Suttles J. (2008). Adenosine 5'-monophosphate-activated protein kinase promotes macrophage polarization to an anti-inflammatory functional phenotype. J. Immunol..

[64] Cao Q., Cui X., Wu R., Zha L., Wang X., Parks J.S., Yu L., Shi H., Xue B. (2016). Myeloid Deletion of α1AMPK Exacerbates Atherosclerosis in LDL Receptor Knockout (LDLRKO) Mice. Diabetes.

[65] Kopp E., Ghosh S. (1994). Inhibition of NF-kappa B by sodium salicylate and aspirin. Science.

[66] Pierce J.W., Read M.A., Ding H., Luscinskas F.W., Collins T. (1996). Salicylates inhibit I kappa B-alpha phosphorylation, endothelial-leukocyte adhesion molecule expression, and neutrophil transmigration. J. Immunol..

[67] Yin M.J., Yamamoto Y., Gaynor R.B. (1998). The anti-inflammatory agents aspirin and salicylate inhibit the activity of I(kappa)B kinase-beta. Nature.

[68] Marra D.E., Liao J.K. (2001). Salicylates and vascular smooth muscle cell proliferation: molecular mechanisms for cell cycle arrest. Trends Cardiovasc. Med..

[69] Núñez L., Valero R.A., Senovilla L., Sanz-Blasco S., García-Sancho J., Villalobos C. (2006). Cell proliferation depends on mitochondrial Ca2+ uptake: inhibition by salicylate. J. Physiol..

[70] Broadfield L.A., Marcinko K., Tsakiridis E., Zacharidis P.G., Villani L., Lally J.S.V., Menjolian G., Maharaj D., Mathurin T., Smoke M. (2019). Salicylate enhances the response of prostate cancer to radiotherapy. Prostate.

[71] Zadra G., Photopoulos C., Tyekucheva S., Heidari P., Weng Q.P., Fedele G., Liu H., Scaglia N., Priolo C., Sicinska E. (2014). A novel direct activator of AMPK inhibits prostate cancer growth by blocking lipogenesis. EMBO Mol. Med..

[72] O'Brien A.J., Villani L.A., Broadfield L.A., Houde V.P., Galic S., Blandino G., Kemp B.E., Tsakiridis T., Muti P., Steinberg G.R. (2015). Salicylate activates AMPK and synergizes with metformin to reduce the survival of prostate and lung cancer cells ex vivo through inhibition of de novo lipogenesis. Biochem. J..

[73] Griss T., Vincent E.E., Egnatchik R., Chen J., Ma E.H., Faubert B., Viollet B., DeBerardinis R.J., Jones R.G. (2015). Metformin Antagonizes Cancer Cell Proliferation by Suppressing Mitochondrial-Dependent Biosynthesis. PLoS Biol..

[74] Villani L.A., Smith B.K., Marcinko K., Ford R.J., Broadfield L.A., Green A.E., Houde V.P., Muti P., Tsakiridis T., Steinberg G.R. (2016). The diabetes medication Canagliflozin reduces cancer cell proliferation by inhibiting mitochondrial complex-I supported respiration. Mol. Metabol..

[75] Inoki K., Zhu T., Guan K.L. (2003). TSC2 mediates cellular energy response to control cell growth and survival. Cell.

[76] Gwinn D.M., Shackelford D.B., Egan D.F., Mihaylova M.M., Mery A., Vasquez D.S., Turk B.E., Shaw R.J. (2008). AMPK phosphorylation of raptor mediates a metabolic checkpoint. Mol. Cell.

[77] Wang W., Xiao Z.D., Li X., Aziz K.E., Gan B., Johnson R.L., Chen J. (2015). AMPK modulates Hippo pathway activity to regulate energy homeostasis. Nat. Cell Biol..

[78] Li Y.H., Luo J., Mosley Y.Y.C., Hedrick V.E., Paul L.N., Chang J., Zhang G., Wang Y.K., Banko M.R., Brunet A. (2015). AMP-Activated Protein Kinase Directly Phosphorylates and Destabilizes Hedgehog Pathway Transcription Factor GLI1 in Medulloblastoma. Cell Rep..

[79] Leonhard W.N., van der Wal A., Novalic Z., Kunnen S.J., Gansevoort R.T., Breuning M.H., de Heer E., Peters D.J.M. (2011). Curcumin inhibits cystogenesis by simultaneous interference of multiple signaling pathways: in vivo evidence from a Pkd1-deletion model. Am. J. Physiol. Renal Physiol..

[80] Dachineni R., Kumar D.R., Callegari E., Kesharwani S.S., Sankaranarayanan R., Seefeldt T., Tummala H., Bhat G.J. (2017). Salicylic acid metabolites and derivatives inhibit CDK activity: Novel insights into aspirin's chemopreventive effects against colorectal cancer. Int. J. Oncol..

[81] Sankaranarayanan R., Valiveti C.K., Dachineni R., Kumar D.R., Lick T., Bhat G.J. (2020). Aspirin metabolites 2,3-DHBA and 2,5-DHBA inhibit cancer cell growth: Implications in colorectal cancer prevention. Mol. Med. Rep..

[82] Zhang C., Balbo B., Ma M., Zhao J., Tian X., Kluger Y., Somlo S. (2021). Cyclin-Dependent Kinase 1 Activity Is a Driver of Cyst Growth in Polycystic Kidney Disease. J. Am. Soc. Nephrol..

[83] Maser R.L., Vassmer D., Magenheimer B.S., Calvet J.P. (2002). Oxidant stress and reduced antioxidant enzyme protection in polycystic kidney disease. J. Am. Soc. Nephrol..

[84] Yin J., Ren W., Huang X., Deng J., Li T., Yin Y. (2018). Potential Mechanisms Connecting Purine Metabolism and Cancer Therapy. Front. Immunol..

[85] Leonardi R., Jackowski S. (2007). Biosynthesis of Pantothenic Acid and Coenzyme A. EcoSal Plus.

[86] Taylor S.L., Ganti S., Bukanov N.O., Chapman A., Fiehn O., Osier M., Kim K., Weiss R.H. (2010). A metabolomics approach using juvenile cystic mice to identify urinary biomarkers and altered pathways in polycystic kidney disease. Am. J. Physiol. Renal Physiol..

[87] Mi Z., Song Y., Cao X., Lu Y., Liu Z., Zhu X., Geng M., Sun Y., Lan B., He C. (2020). Super-enhancer-driven metabolic reprogramming promotes cystogenesis in autosomal dominant polycystic kidney disease. Nat. Metab..

[88] Browne G.J., Finn S.G., Proud C.G. (2004). Stimulation of the AMP-activated protein kinase leads to activation of eukaryotic elongation factor 2 kinase and to its phosphorylation at a novel site, serine 398. J. Biol. Chem..

[89] Lantinga-van Leeuwen I.S., Leonhard W.N., van der Wal A., Breuning M.H., de Heer E., Peters D.J.M. (2007). Kidney-specific inactivation of the Pkd1 gene induces rapid cyst formation in developing kidneys and a slow onset of disease in adult mice. Hum. Mol. Genet..

[90] Dieterle F., Ross A., Schlotterbeck G., Senn H. (2006). Probabilistic quotient normalization as robust method to account for dilution of complex biological mixtures. Application in 1H NMR metabonomics. Anal. Chem..

[91] Leonhard W.N., Kunnen S.J., Plugge A.J., Pasternack A., Jianu S.B.T., Veraar K., El Bouazzaoui F., Hoogaars W.M.H., Ten Dijke P., Breuning M.H. (2016). Inhibition of Activin Signaling Slows Progression of Polycystic Kidney Disease. J. Am. Soc. Nephrol..

[92] Wang H., Robinson J.L., Kocabas P., Gustafsson J., Anton M., Cholley P.E., Huang S., Gobom J., Svensson T., Uhlen M. (2021). Genome-scale metabolic network reconstruction of model animals as a platform for translational research. Proc. Natl. Acad. Sci. USA.

[93] Hastings J., Owen G., Dekker A., Ennis M., Kale N., Muthukrishnan V., Turner S., Swainston N., Mendes P., Steinbeck C. (2016). ChEBI in 2016: Improved services and an expanding collection of metabolites. Nucleic Acids Res..

[94] Frankish A., Diekhans M., Ferreira A.-M., Johnson R., Jungreis I., Loveland J., Mudge J.M., Sisu C., Wright J., Armstrong J. (2019). GENCODE reference annotation for the human and mouse genomes. Nucleic Acids Res..

[95] Patil K.R., Nielsen J. (2005). Uncovering transcriptional regulation of metabolism by using metabolic network topology. Proc. Natl. Acad. Sci. USA.

