# Supplementary material for: Short salsalate administration affects cell proliferation, metabolism, and inflammation in polycystic kidney disease
Source: iScience. 2023 Oct 19;26(11):108278. doi: 10.1016/j.isci.2023.108278 (PMC10665819; doi:10.1016/j.isci.2023.108278)
Supplement: Document S1. Figures S1–S7 and Tables S3 and S10 [file mmc1.pdf]

iScience, Volume *26*

Supplemental information

Short salsalate administration affects cell proliferation, metabolism, and inflammation in polycystic kidney disease

Anish A. Kanhai, Elena Sánchez-López, Thomas B. Kuipers, Jan B. van Klinken, Kyra L. Dijkstra, Inge van der Veen, Hans J. Baelde, Xuewen Song, York Pei, Hailiang Mei, Wouter N. Leonhard, Oleg A. Mayboroda, and Dorien J.M. Peters

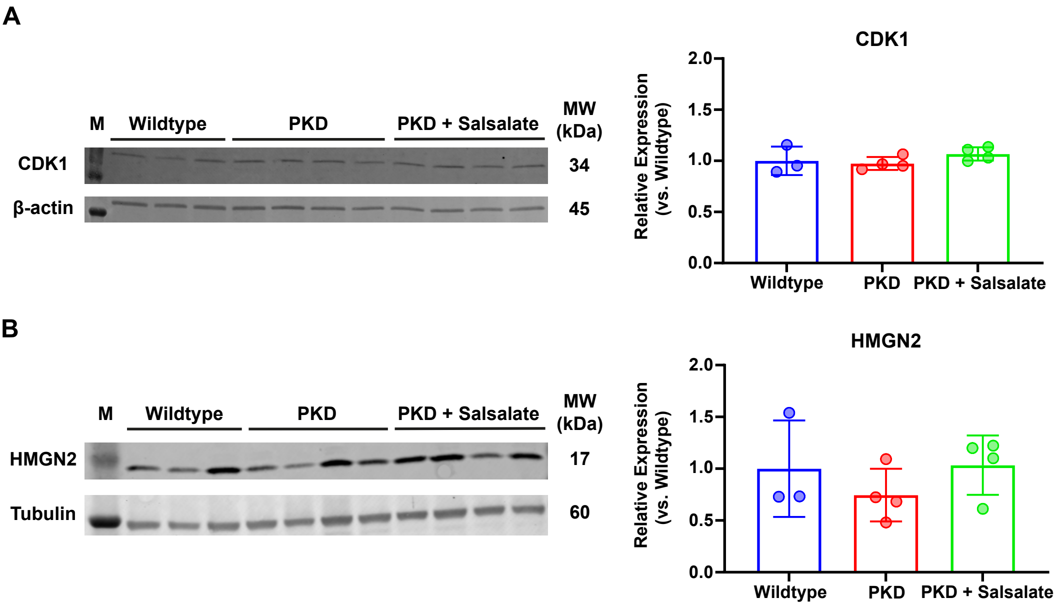

Figure S1: Short salsalate treatment does not affect protein expression of RNA-sequencing hits CDK1 and HMGN2 (related to Figure 6)

(A) Western blotting for CDK1 on protein extracts from wildtype, PKD and PKD + SAL animals. β-actin protein expression was used as an internal loading control. Quantification of the CDK1 relative to β-actin. No significant differences were detected between any of the three groups. **(B)** Western blotting for HMGN2 on protein extracts from wildtype, PKD and PKD + SAL animals. Tubulin protein expression was used as an internal loading control. Quantification of HMGN2 relative to tubulin. No significant differences were detected between any of the three groups. Each dot represents a mouse kidney (n = 3-4 animals per group). Data presented are mean ± SD. M = marker, MW = molecular weight, WT = wildtype, SAL = Salsalate, kDa = kilodalton.

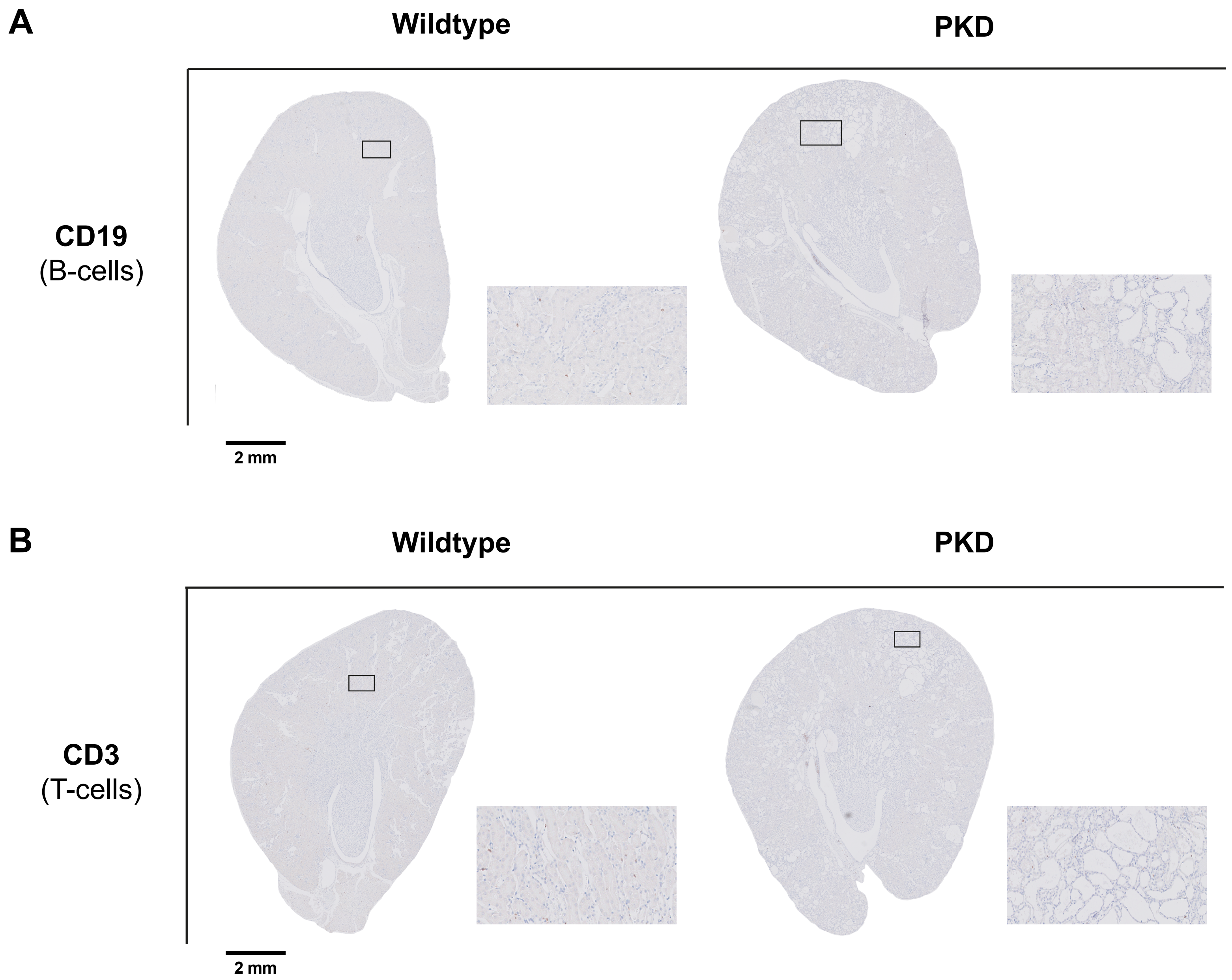

Figure S2: No differences in B- and T-cell presence between PKD and wildtype kidneys (related to Figure 6)
(A) Immunohistochemical staining for the B-cell marker CD19, which shows a very low B-cell presence in both PKD and wildtype mice, and no differences between both groups. **(B)** Immunohistochemical staining for the T-cell marker CD3, which shows a very low T-cell presence in both PKD and wildtype mice, and no differences between both groups. Scalebar = 2 mm.

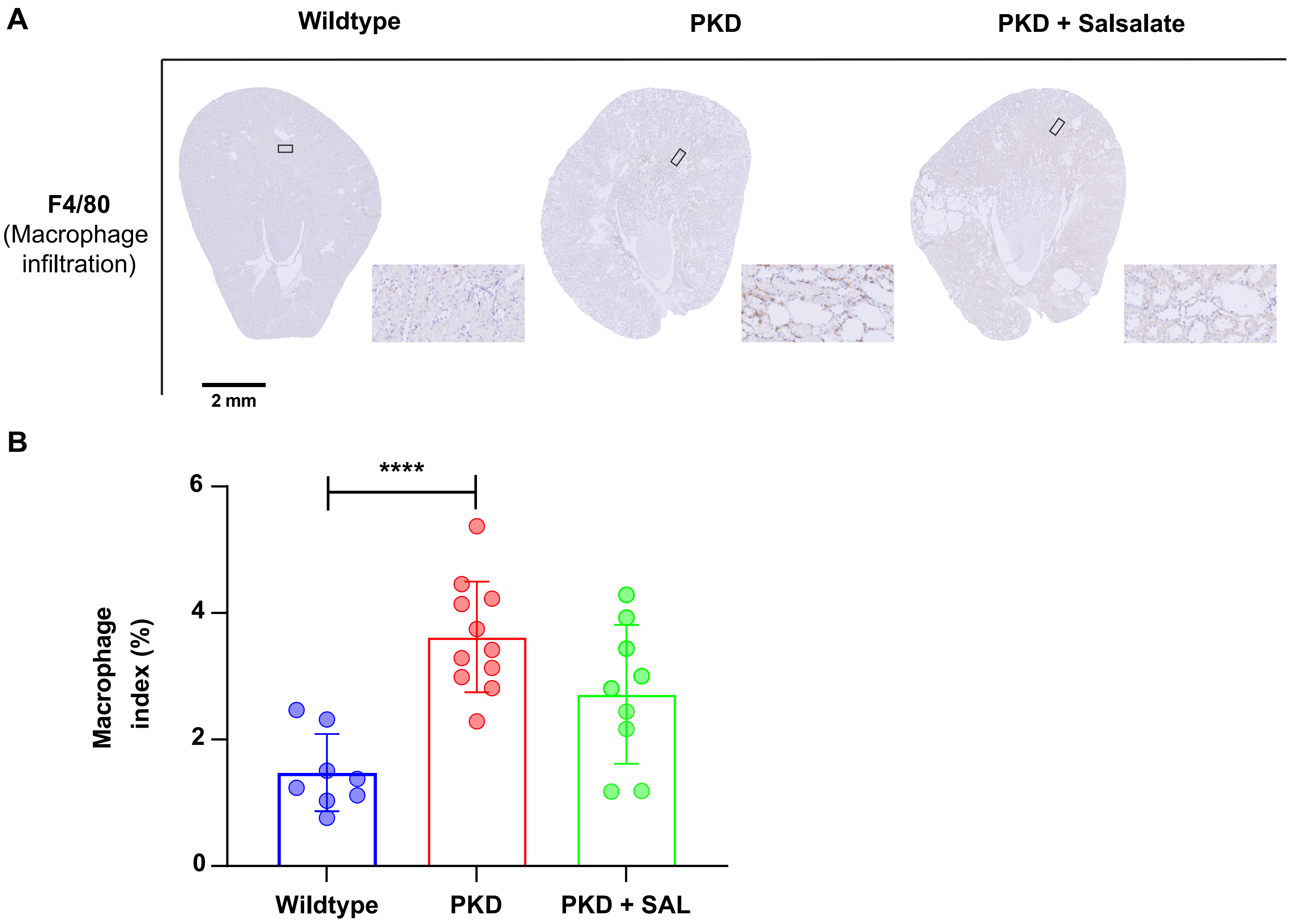

Figure S3: Short salsalate treatment has no significant effects on macrophage infiltration (related to Figure 6)
(A) Immunohistochemical staining for the macrophage infiltration marker F4/80, which shows no differences between PKD + Salsalate mice and PKD mice. **(B)** Quantification of F4/80 staining. PKD animals have significantly more macrophage infiltration (measured by % positive pixels over total amount of pixels) compared to wildtype animals. No difference between PKD + Salsalate and PKD animals was detected. Data presented are mean ± SD. Each dot represents a mouse kidney. Scalebar = 2 mm. ****P < 0.0001, measured by two-way unpaired Student's t-test. WT = wildtype, SAL = salsalate.

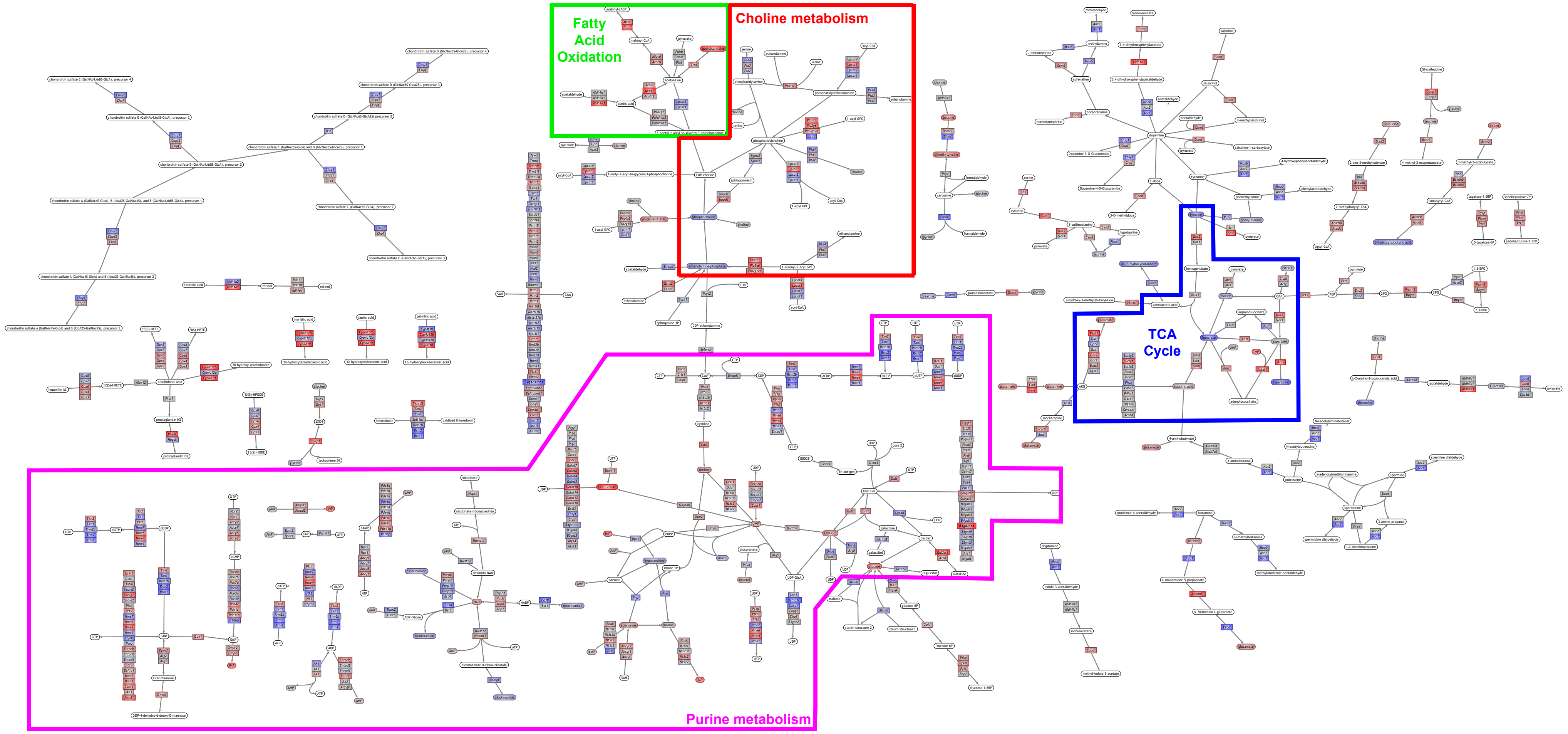

Figure S4: Visual map of short salsalate treatment-induced changes in PKD (related to Figure 8)
Integrated visual representation of metabolic and transcriptomic changes, comparing PKD + SAL animals to PKD animals. Metabolic processes of interest have been high-lighted in the figure (green = fatty acid oxidation, red = choline metabolism, blue = TCA cycle, purple = purine metabolism), magnifications are presented in Figures S5-S7. Red shaded metabolites/genes are increased in PKD + SAL, blue shaded metabolites/genes are decreased in PKD + SAL. White shaded metabolites were not measured, but are part of the displayed reaction(s). Color intensity reflects the degree of change. Rounded frames include metabolites, boxed frames include genes. Metabolites/genes in bold are significantly different. Maps were created with the MRN pathway browser tool (see STAR Methods) and can be visited at https://lumc.github.io/MRN-browser/salsalate-multiomics.

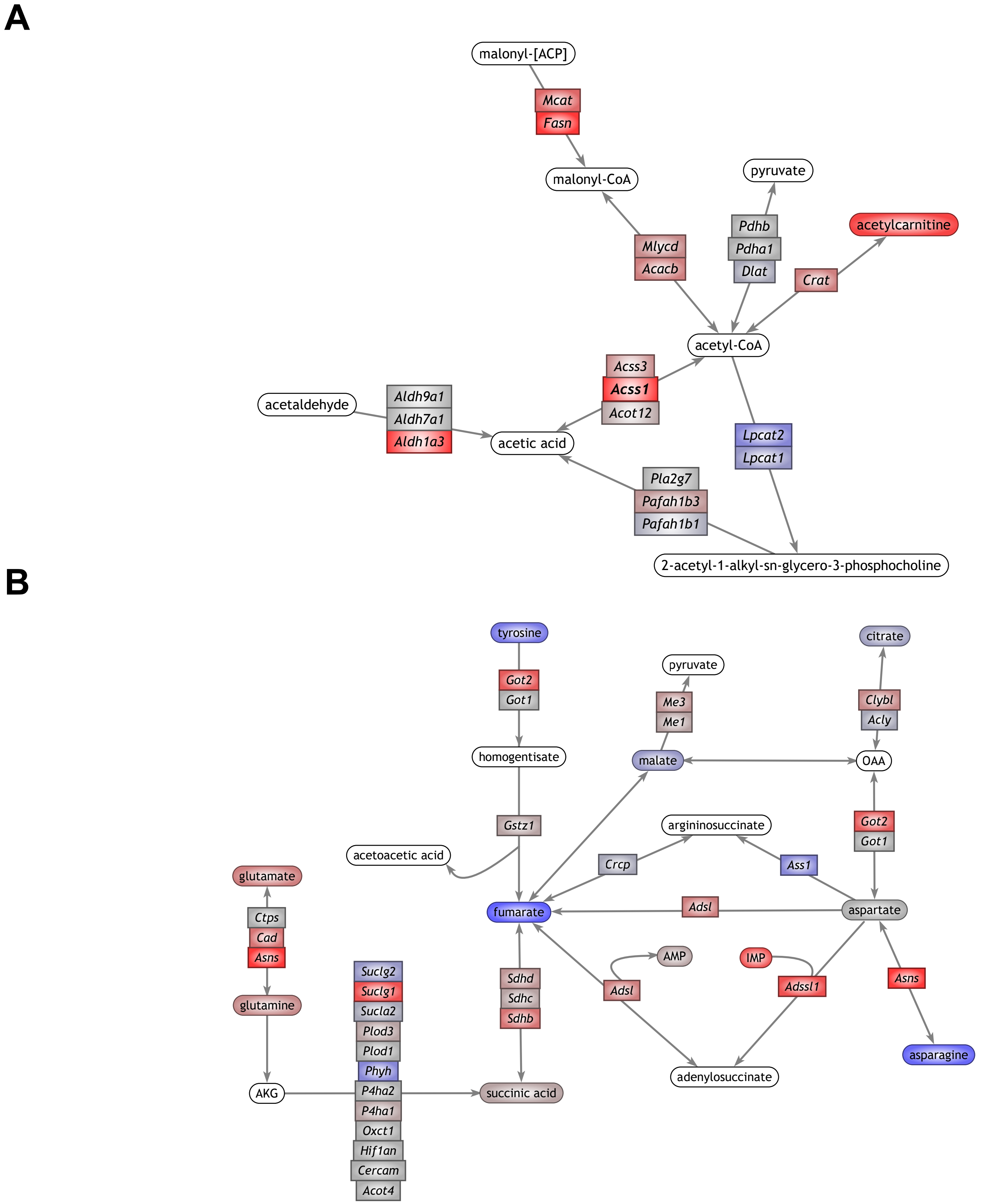

Figure S5: Visual map of fatty acid oxidation and TCA cycle changes induced by salsalate treatment in PKD (related to Figure 8)
Visual representation of both metabolic and transcriptomic changes related to **(A)** fatty acid oxidation and **(B)** the TCA cycle, comparing PKD + SAL animals to PKD animals. Both panels are zoomed in cut-outs from Figure S4. Note that some elements might have been moved compared to Figure S4, for clearer visualization. Red shaded metabolites/genes are increased in PKD + SAL, blue shaded metabolites/genes are decreased in PKD + SAL. White shaded metabolites were not measured, but are part of the displayed reaction(s). Color intensity reflects the degree of change. Rounded frames include metabolites, boxed frames include genes. Metabolites/genes in bold are significantly different. Maps were created with the MRN pathway browser tool (see STAR Methods).

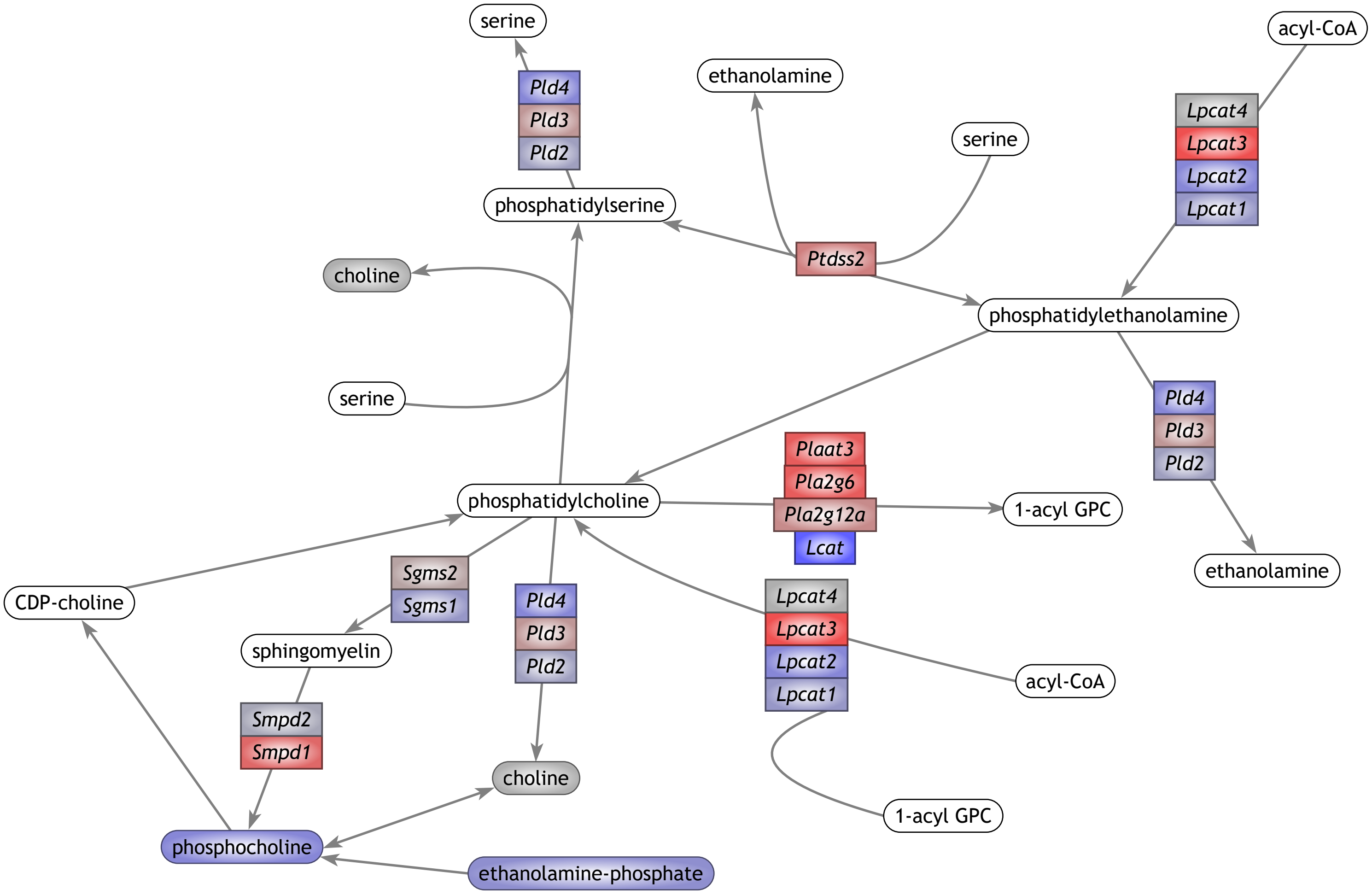

Figure S6: Visual map of choline metabolism changes induced by salsalate treatment in PKD (related to Figure 8)
Visual representation of both metabolic and transcriptomic changes related to choline metabolism, comparing PKD + SAL animals to PKD animals. This figure is a zoomed in cut-out from Figure S4. Note that some elements might have been moved compared to Figure S4, for clearer visualization. Red shaded metabolites/genes are increased in PKD + SAL, blue shaded metabolites/genes are decreased in PKD + SAL. White shaded metabolites were not measured, but are part of the displayed reaction(s). Color intensity reflects the degree of change. Rounded frames include metabolites, boxed frames include genes. Metabolites/genes in bold are significantly different. Maps were created with the MRN pathway browser tool (see STAR Methods).

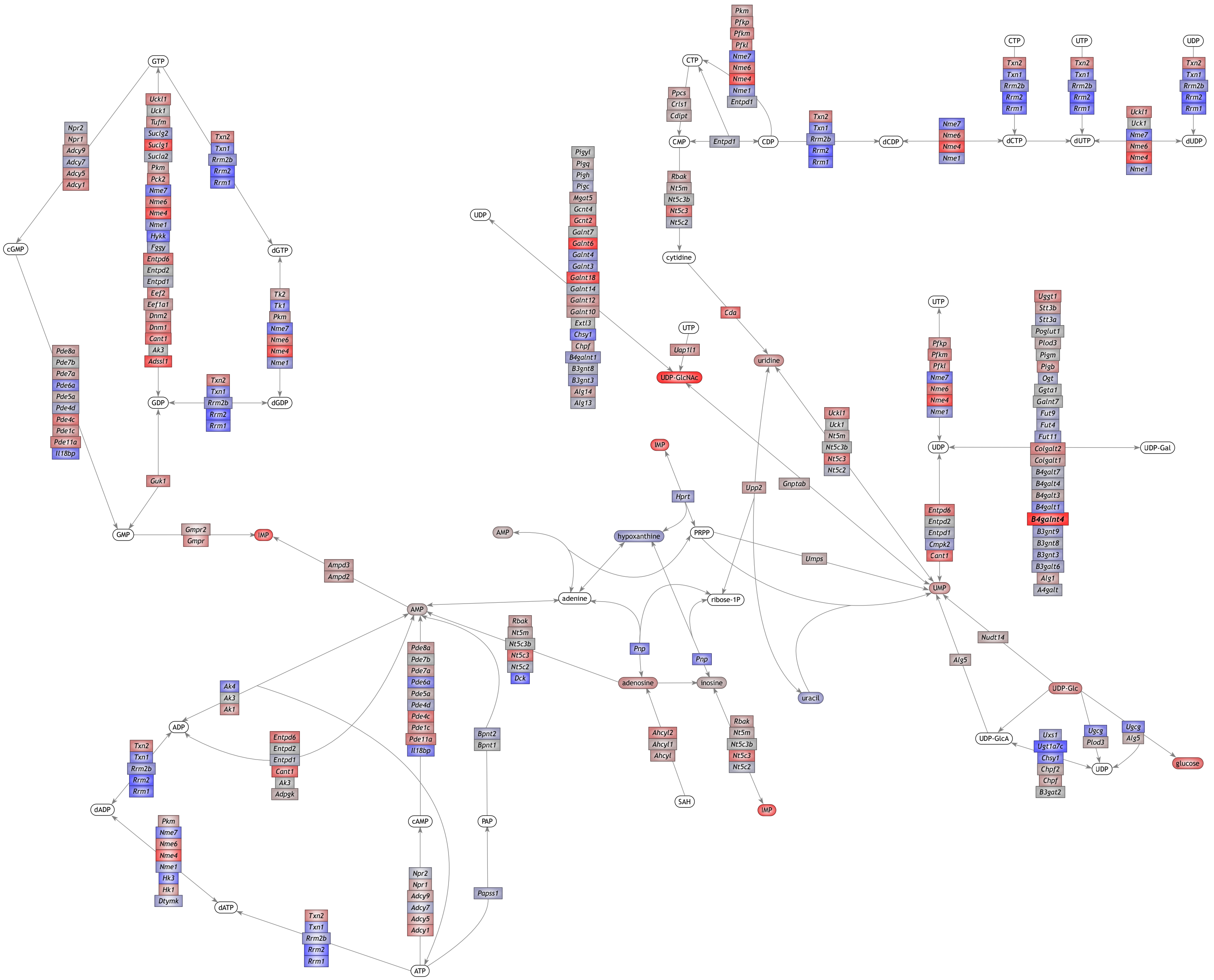

Figure S7: Visual map of purine metabolism changes induced by salsalate treatment in PKD (related to Figure 8)

Visual representation of both metabolic and transcriptomic changes related to purine metabolism, comparing PKD + SAL animals to PKD animals. This figure is a zoomed in cut-out from Figure S4. Note that some elements might have been moved compared to Figure S4, for clearer visualization. Red shaded metabolites/genes are increased in PKD + SAL, blue shaded metabolites/genes are decreased in PKD + SAL. White shaded metabolites were not measured, but are part of the displayed reaction(s). Color intensity reflects the degree of change. Rounded frames include metabolites, boxed frames include genes. Metabolites/genes in bold are significantly different. Maps were created with the MRN pathway browser tool (see STAR Methods).

Table S3 – Comparison of two kidney weight/body weight ratio (2KW/BW%) of mice from the long salsalate treatment and short salsalate treatment study, used for analysis (related to Figure 1 & 2)

Long salsalate treatment			Short salsalate treatment		
Wildtype	PKD	PKD + Salsalate	Wildtype	PKD	PKD + Salsalate
0.950656406	2.296521445	1.81680545	1.149425287	2.556325823	2.591128678
1.017811705	2.352941176	1.854599407	1.260504202	2.60756193	2.745098039
1.063394683	2.463222716	1.902455898	1.32038835	2.69541779	2.846975089
1.098484848	2.539802881	1.968951155	1.342008947	2.825607064	2.852128126
1.104536489	2.610669694	2.013189865	1.387720774	2.965328467	2.948504983
1.10729286	2.8515625	2.082529888	1.396303901	3.139991278	3.041647169
1.185921959	2.92184076	2.125193199	1.469353484	3.248463565	3.107466552
1.262916188	3.130823705	2.278769464	1.528294093	3.350739774	3.173851255
	4.061895551	2.489177489		3.360215054	3.242677824
	4.342723005	2.50829952		3.42405619	3.569910752
	4.837398374	2.539208364		3.470031546	3.638104356
	4.87704918	2.886836028		3.630658897	3.870106762
	5.398550725	2.991452991			
	5.593607306	3.492573264			
	5.794460641	3.887850467			
	5.916030534	4.710772428			
	6.643757159	5.314371257			
	6.871932173	5.596211795			
	6.926586479	6.110684089			
	7.547169811	6.25698324			
	8.221024259	9.867256637			
	8.609865471				
	9.581589958				
	10.85991118				
	11.96261682				
	12.08791209				

Table S10: Primer sequences used for described qPCR experiments (related to Figure 6 & STAR Methods)

Gene name	Forward primer sequence (5' → 3')	Reverse primer sequence (5' → 3')
Acss1	AGCCTATCAACCACGAAGCC	AGATGCCTCCAGTTTCCGTT
Cdc20	GAATGCCCCAGAAGGCTACC	AGTCATTCCGGATTTCGGGG
Cdca8	ATGGCTCCCAAGAAACGCAG	TGGTTCGAACTTGCACCTCG
Chek1	GAGGGAAGGCCATATCCAGTATC	GCATCCCTATGTCTGGCTCAA
Cxcl10	ACCATGAACCCAAGTGCTGC	GCTTCCCTATGGCCCTCATTC
Depp1	TCTAGCTCCCACAATGCGAC	GGTGGTCACTCCACCTACAC
Ftcd	CGTTTGCTGCCTGTTTGGAG	ACAGTCTCGGCCAGTTTCAG
Hprt	GGCTATAAGTTCTTTGCTGACCTG	AACTTTTATGTCCCCCGTTGA
Il1rn	GGAAGGCAGTGGAAGACCTTG	GCAGGGTCTTTTCCCAGAAGG
Mcm2	ACAGAGCCCATCATTTCCCG	GGCCAGCATCTCATCCTGAA
Vnn1	GCATGCTGTGATCCTGCCTA	ATAATGTGCGCACCCTGCTT
